# Safety and efficacy of fecal microbiota transplantation for autoimmune diseases and autoinflammatory diseases: A systematic review and meta-analysis

**DOI:** 10.3389/fimmu.2022.944387

**Published:** 2022-09-30

**Authors:** Liuting Zeng, Ying Deng, Kailin Yang, Junpeng Chen, Qi He, Hua Chen

**Affiliations:** ^1^ Department of Rheumatology and Clinical Immunology, Peking Union Medical College Hospital, Chinese Academy of Medical Sciences & Peking Union Medical College, National Clinical Research Center for Dermatologic and Immunologic Diseases (NCRC-DID), Key Laboratory of Rheumatology and Clinical Immunology, Ministry of Education, Beijing, China; ^2^ People's Hospital of Ningxiang City, Ningxiang City, China; ^3^ Key Laboratory of Hunan Province for Integrated Traditional Chinese and Western Medicine on Prevention and Treatment of Cardio-Cerebral Diseases, Hunan University of Chinese Medicine, Changsha, China; ^4^ School of Mechanical Engineering, Hunan University of Science and Technology, Xiangtan, China

**Keywords:** fecal microbiota transplantation, autoimmune diseases, autoinflammatory diseases, meta-analysis, systemic review

## Abstract

**Objective:**

To evaluate the safety and efficacy of fecal microbiota transplantation for autoimmune diseases and autoinflammatory diseases.

**Methods:**

Relevant literature was retrieved from the PubMed database, Embase database, Cochrane Library database, etc. The search period is from the establishment of the database to January 2022. The outcomes include clinical symptoms, improvement in biochemistry, improvement in intestinal microbiota, improvement in the immune system, and adverse events. Literature screening and data extraction were independently carried out by two researchers according to the inclusion and exclusion criteria, and RevMan 5.3 software was used for statistics and analysis.

**Results:**

Overall, a total of 14 randomized controlled trials (RCTs) involving six types of autoimmune diseases were included. The results showed the following. 1) Type 1 diabetes mellitus (T1DM): compared with the autologous fecal microbiota transplantation (FMT) group (control group), the fasting plasma C peptide in the allogenic FMT group at 12 months was lower. 2) Systemic sclerosis: at week 4, compared with one of two placebo controls, three patients in the experimental group reported a major improvement in fecal incontinence. 3) Ulcerative colitis, pediatric ulcerative colitis, and Crohn’s disease: FMT may increase clinical remission, clinical response, and endoscopic remission for patients with ulcerative colitis and increase clinical remission for patients with Crohn’s disease. 4) Psoriatic arthritis: there was no difference in the ratio of ACR20 between the two groups.

**Conclusion:**

Based on current evidence, the application of FMT in the treatment of autoimmune diseases is effective and relatively safe, and it is expected to be used as a method to induce remission of active autoimmune diseases.

**Systematic review registration:**

https://www.crd.york.ac.uk/prospero/display_record.php?ID=CRD42021235055, identifier CRD42021235055.

## 1 Introduction

Autoimmune disease refers to a class of diseases caused by the breakdown of the immune system’s immune tolerance to its own components, thereby attacking its own organs, tissues, or cells, causing damage (1, 2). According to the scope of involved organs and tissues, autoimmune diseases are divided into two categories: organ-specific autoimmune diseases and non-organ-specific autoimmune diseases (3). Organ-specific autoimmune disease refers to lesions confined to a specific organ or tissue, such as type 1 diabetes mellitus (T1DM) (4). Non-organ-specific autoimmune disease refers to a group of diseases with lesions involving multiple tissues, organs, or systems, including systemic lupus erythematosus (SLE) and rheumatoid arthritis (RA) (5). Epidemiology shows that about 7.6% to 9.4% of the global population suffer from various types of autoimmune diseases. Autoimmune diseases have become the third largest chronic disease after cardiovascular disease and cancer (6, 7). Autoimmune diseases are difficult to cure and may seriously affect the quality of life of patients and threaten the lives of patients (8).

At present, the treatment of autoimmune disease is mainly symptomatic therapy (inhibition of inflammation and inhibition of autoimmunity) (9, 10). For example, the main drugs for RA are conventional synthetic disease-modifying anti-rheumatic drugs (csDMARDs). After disease remission, a reasonable reduction of csDMARDs is closely related to the risk of disease recurrence (11, 12). Biologics DMARDs (bDMARDs) are commonly used to treat patients with moderate-to-severe rheumatic disease who do not respond well to or cannot tolerate conventional synthetic DMARDs (12, 13). However, none of these drugs or surgical treatments can completely cure autoimmune diseases, only relieve symptoms, they have side effects (severe gastrointestinal adverse reactions and immunosuppression leading to infection), and the price of biological agents is high (14–16). With the deepening of research, there is evidence that the intestinal microbiota can maintain the body’s homeostasis. It has a certain effect on the regulation of cytokines, helps to improve the defense ability of the body’s immune system, and can participate in the host’s immune process (17, 18). The gut microbiota has been shown to interact with immune cells and modulate specific signaling pathways involved in innate and adaptive immune processes (19). Current research shows that gut microbes are closely related to diseases (20), especially in autoimmune diseases (21). A number of animal experiments and clinical trials have found that intestinal microbiota may become a new therapy for the treatment of autoimmune diseases (21, 22).

Fecal microbiota transplantation (FMT) refers to transplanting the functional microbiota in the feces of healthy people into the patient’s intestine to rebuild the healthy intestinal microbiota and achieve the treatment of intestinal and extraintestinal diseases (23), especially for the treatment of *Clostridium difficile* infection. It has become an important treatment method recommended by US medical guidelines (24). FMT is not just a simple technology but an emerging treatment that includes strict donor screening, optimized fecal bacteria preparation methods, scientific microbiota transplantation methods, and other concepts and methodologies (25). A meta-analysis summarized data from 26 studies and found that FMT can significantly relieve ulcerative colitis (UC), and has some effect on liver disease, metabolic syndrome/obesity, and antibiotic resistance but has no significant effect on irritable bowel syndrome. In addition, fecal microbiota transplantation showed a good safety profile relative to the control group (26). A number of randomized controlled trials (RCTs) have found that the efficacy of FMT in the treatment of autoimmune diseases is significantly better than that of conventional treatment, and no serious adverse reactions have been observed during follow-up (27–30). Combining the results of the current clinical studies, we consider that FMT may be a potential option for professional physicians in the treatment of autoimmune diseases. However, due to the differences in the transplantation route, preparation process, and follow-up time of FMT among the studies, there may be some differences in the results, and the sample size of each study is relatively small. Therefore, this study conducted a meta-analysis of all RCTs on FMT in the treatment of autoimmune diseases so far and comprehensively evaluate its efficacy and safety so as to provide an evidence-based basis for guiding its clinical application.

## 2 Materials and methods

### 2.1 Research proposal

This study was performed in strict accordance with the Preferred Reporting Items for Systematic Reviews and Meta-Analyses (PRISMA) guidelines and the protocol registered in PROSPERO (CRD42021235055) (see **Supplementary Material**).

### 2.2 Selection criteria

#### 2.2.1 Participants

The participants were adult patients with autoimmune diseases that are clearly diagnosed by recognized standards.

#### 2.2.2 Intervention

The experimental group was given any method of FMT treatment, not limited to transplantation methods. The control group received non-FMT treatments, such as conventional therapies.

#### 2.2.3 Outcomes

The outcomes include improvement in clinical symptoms, improvement in biochemistry, improvement in intestinal microbiota, improvement in the immune system, and adverse events.

#### 2.2.4 Study design

RCTs, with no limitation on language, random sequence generation methods, etc., were included.

#### 2.2.5 Exclusion criteria

1) Interventions in the control group included FMT. 2) Duplicate literature: research was conducted in the same center, and duplicate cases were excluded after the full text was read; if the article was duplicated in Chinese and English, the latest published literature was selected. The following was also excluded: 3) non-RCTs, 4) non-adult patients, and 5) non-clinical research such as animal experiments, cytology research, or molecular biology research.

### 2.3 Search strategy

The researchers searched PubMed, CNKI, Wanfang Database, Web of Science, VIP Database Medline Complete, Sinomed, and Embase for RCTs on FMT in the treatment of autoimmune diseases. The retrieval time was from inception to 16 January 2022. The Cochrane Library (to Issue 1, 2022), China Clinical Trial Registry, and ClinicalTrials.gov were also searched. The search strategy is shown in **Table S1** as an example.

### 2.4 Data extraction and quality assessment

Literature screening and retrieval were independently completed by two researchers according to the inclusion and exclusion criteria. If there was a disagreement, it would be resolved through discussion with all researchers. The following data were extracted for the included studies: author, publication year, study type, total number of study cases, number of patients included, average age, source of stool, transplantation method, relevant outcomes, adverse reactions and cases, and follow-up time. The included RCT literature was scored according to the Cochrane Risk Bias Assessment Tool (31), and the scoring was completed by two investigators independently. If there is a disagreement, it will be resolved through discussion with all researchers.

### 2.5 Statistical analysis

Review Manager 5.3 was used in the software for meta-analysis (32). The Q test is used to test the heterogeneity between the results of the included studies, and combined with I^2^ to quantitatively analyze the magnitude of the heterogeneity. If p > 0.1 and I^2^ < 50%, then the heterogeneity between the results of each study is considered to be small, and the fixed-effects model was used for meta-analysis; otherwise, the subgroup analysis is performed first, and if there is still heterogeneity, the random-effects model was used for meta-analysis. The risk ratio (RR) and its 95% confidence interval (CI) were used as the efficacy and safety statistics (33).

## 3 Results

### 3.1 Results of the search

**Figure 1:**
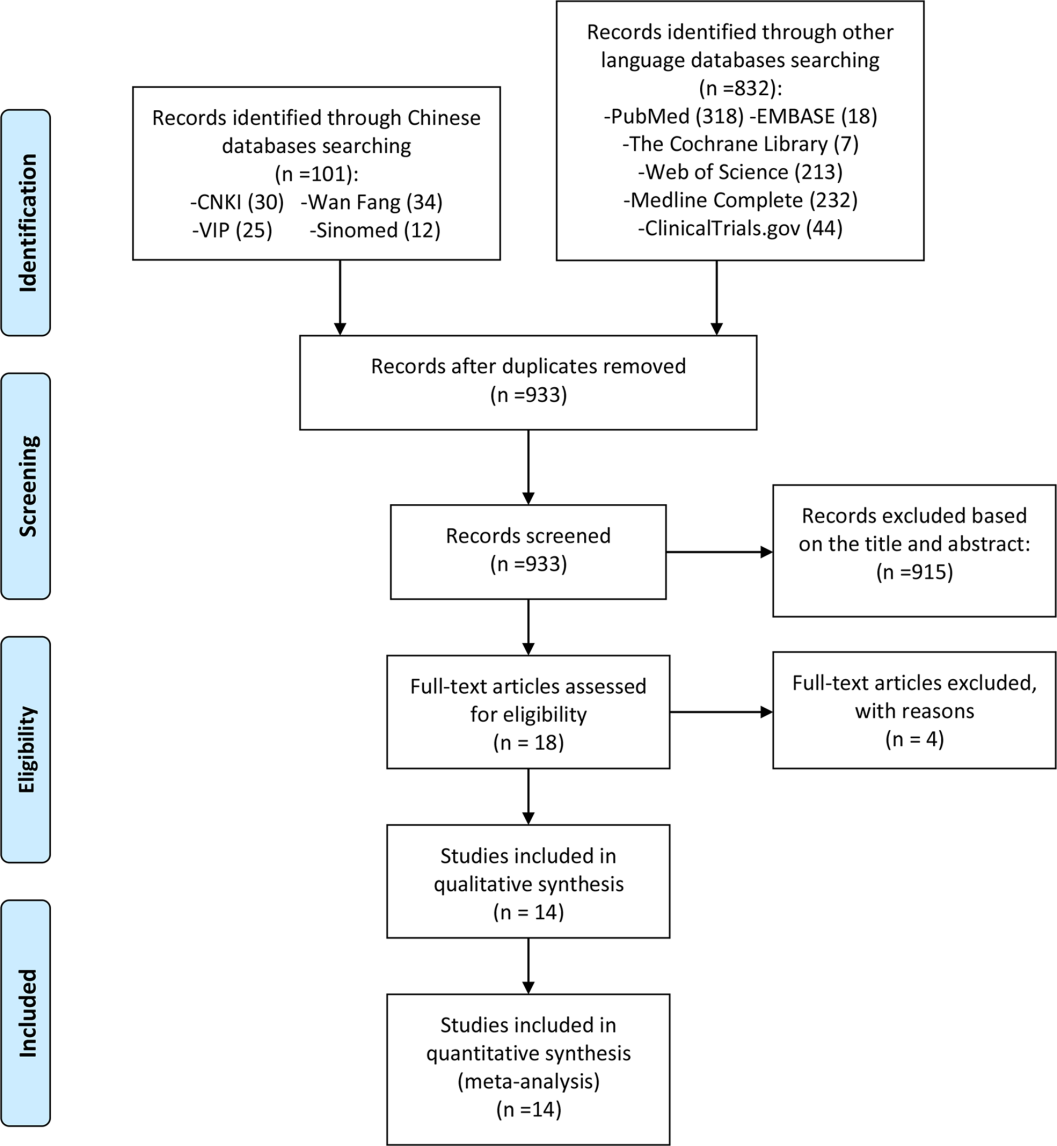
Flow diagram.

After a preliminary search, a total of 933 records were retrieved. Then 18 articles were initially included according to the search strategy. After reading the full text carefully and comparing the inclusion and exclusion criteria, four RCTs were excluded (34–39), and 14 RCTs were finally included (40–53) (**Figure 1**).

### 3.2 Description of included trials

Fourteen RCTs involving six types of autoimmune diseases were included: T1DM (one RCT), systemic sclerosis (one RCT), ulcerative colitis (eight RCTs), pediatric ulcerative colitis (one RCT), Crohn’s disease (two RCTs), and psoriatic arthritis (one RCT). Most of the RCTs described the clinical research registration numbers. In addition, no more than 100 participants were involved in the endpoint assessment. Most studies performed FMT through gastroscopy or colonoscopy, four of which were performed through the upper gastrointestinal tract, and eight were performed through the lower gastrointestinal tract, while Haifer et al. (2022) (48) and Crothers et al. (2022) (49) used oral lyophilized FMT. The source of fecal bacteria of Fretheim et al. (2020) (41) is standardized human fecal microbiota composition, which originates from a single healthy feces donor in 1995. Sun et al. (2018) (46) did not mention the source of fecal bacteria. The fecal bacteria used in the remaining RCTs are from healthy pre-screened donors, and the fecal bacteria used by different patients come from different donors. The details of the study characteristics are presented in **Table 1**.

**Table 1:** The characteristics of the included studies.

Disease	Study	Trial registration number	Country	Sample size		Intervention		Relevant outcomes	Mean age (years)		Duration	Source of fecal microbiota
				Trial group	Control group	Trial group	Control group		Trial group	Control group		
T1DM	de Groot et al. (2021) (40)	NTR3697	Netherlands	17	17	Allogenic FMT to upper gastrointestinal tract	Autologous FMT to upper gastrointestinal tract	C peptide and HbA1c, T-cell immunology changes, fecal microbiota changes, plasma metabolite changes upon FMT, adverse events	25.0 ± 3.5	24.3 ± 5.4	48 weeks	Lean (BMI < 25 kg/m^2^), omnivorous, healthy male and female Caucasians
Systemic sclerosis	Fretheim et al. (2020) (41)	NCT03444220	Norway	5	5	Commercially available anaerobic cultivated human intestinal microbiota (ACHIM) transplant to upper gastrointestinal tract	ACHIM bacteria medium transplant to upper gastrointestinal tract	Clinical symptoms, modified Rodnan Skin Score (mRSS), new-onset digital ulcers, forced vital capacity (FVC), diffusing capacity of the lungs for carbon monoxide (DLCO), CRP, ESR, fecal microbiota changes, adverse events	58.0 ± 5.6	66.0 ± 1.5	16 weeks	ACHIM is produced by ACHIM AB biotherapeutics (556939-7788), Sweden. It is a standardized human fecal microbiota composition. The microbiota originates from feces donated back in 1995, by a single healthy feces donor
Ulcerative colitis	Paramsothy et al. (2017) (42)	NCT01896635	Australia	41	40	FMT to lower gastrointestinal tract	Normal saline transfer to lower gastrointestinal tract	Clinical remission, clinical response, endoscopic remission, endoscopic response, adverse event	27.8–48.9	27.7–45.6	8 weeks	Healthy anonymous pre-screened donors
	Rossen et al. (2015) (43)	NCT01650038	Netherlands	23	25	Allogenic FMT to upper gastrointestinal tract	Autologous FMT to upper gastrointestinal tract	Clinical remission, clinical response, endoscopic remission, endoscopic response, adverse event	33.0–56.0	30.0–48.0	12 weeks	Healthy partners, relatives, or volunteers (≥18 years of age)
	Moayyedi et al. (2015) (44)	NCT01545908	Canada	38	37	FMT to lower gastrointestinal tract	Water enema transfer to lower gastrointestinal tract	Clinical remission, clinical response, adverse event	28–52	35.8 ± 12.1	7 weeks	Volunteers who were between 18 and 60 years of age and were otherwise healthy, as assessed by a screening questionnaire
	Costello et al. (2019) (45)	ACTRN12613000236796	Australia	38	35	Allogenic FMT to lower gastrointestinal tract	Autologous FMT to lower gastrointestinal tract	Clinical remission, clinical response, endoscopic remission, adverse event	42.2 ± 15.0	25–46	8 weeks	Healthy anonymous pre-screened donors
	Sun et al. (2018) (46)	No registration information found	China	14	15	FMT to lower gastrointestinal tract + oral mesalazine 1 g t.i.d.	Normal saline transfer to lower gastrointestinal tract + oral mesalazine 1 g t.i.d.	Clinical remission, adverse events	52.64 ± 13.91	43.6 ± 13.87	16 weeks	Not mentioned
	Deng et al. (2020) (47)	No registration information found	China	24	10	FMT to lower gastrointestinal tract + oral mesalazine 1 g q.i.d.	Oral mesalazine 1 g q.i.d.	Adverse events	39.5	42	8 weeks	Healthy children or adolescents from 6 to 15 years old and meet the following conditions: 1) there is no known infectious disease and no antibacterial drugs have been used within 3 months; 2) no gastrointestinal tumors, polyps, and other diseases; 3) no history of immune system diseases; no immunosuppressive agents have been used; 4) no history of IBD, chronic constipation, or IBS; no history of malignant tumor; 5) have not traveled to areas with endemic diarrhea in the last 6 months; 6) and there are no digestive system symptoms and other related risk factors, such as intravenous drug abuse (drug abuse), high-risk sexual behavior, and criminal history. Donor exclusion criteria: 1) metabolic diseases such as diabetes and metabolic syndrome; 2) history of digestive system surgery; 3) chronic fatigue syndrome; 4) autoimmune diseases; 5) atopic diseases, such as eczema, asthma, and gastrointestinal eosinophil-related diseases; and 6) neuropsychiatric diseases
	Haifer et al. (2022) (48)	ACTRN12619000611123	Australia	15	20	Oral lyophilized FMT	Oral placebo	Clinical remission, endoscopic remission, adverse event	31.8–46.8	25.1–42.0	8 weeks	Healthy unrelated donors
	Crothers et al. (2022) (49)	NCT02390726	the U.S.	6	6	Oral lyophilized FMT	Oral placebo	Clinical remission, adverse events	41 ± 15	52 ± 15	12 weeks	Healthy unrelated donors
Crohn’s disease	Sokol et al. (2020) (50)	NCT02097797	France	8	9	FMT to lower gastrointestinal tract	Sham FMT	Clinical remission, adverse events	27.5–36.5	33.0–52.0	10 weeks	Healthy pre-screened donors
	Sood et al. (2019) (51)	CTRI/2018/02/012148	India	31	30	FMT to lower gastrointestinal tract	Normal saline transfer to lower gastrointestinal tract	Clinical remission, Endoscopic remission, adverse events	33 ± 12.4	34.6 ± 12.3	48 weeks	Healthy pre-screened donors
Pediatric Ulcerative Colitis	Pai et al. (2021) (52)	No registration information found	Canada	19	12	FMT to lower gastrointestinal tract	Placebo enema transfer to lower gastrointestinal tract	Clinical remission, Endoscopic remission, adverse events	4–17		144 weeks	Healthy pre-screened donors
Psoriatic arthritis	Kragsnaes et al. (2021) (53)	NCT03058900	Denmark	15	16	FMT to upper gastrointestinal tract	Sham FMT	Health Assessment Questionnaire Disability Index (HAQ-DI), Health Assessment Questionnaire Disability Index (ACR)20, Spondyloarthritis Research Consortium of Canada (SPARCC) Enthesitis Index, adverse events	48.9 ± 16.1	52.4 ± 11.0	26 weeks	Healthy pre-screened donors

T1DM, type 1 diabetes mellitus; FMT, fecal microbiota transplantation; HbA1c, glycated hemoglobin; BMI, body mass index; CRP, C-reactive protein; ESR, erythrocyte sedimentation rate; IBD, inflammatory bowel disease; IBS, irritable bowel syndrome.

### 3.3 Risk of bias assessment

**Figure 2:**
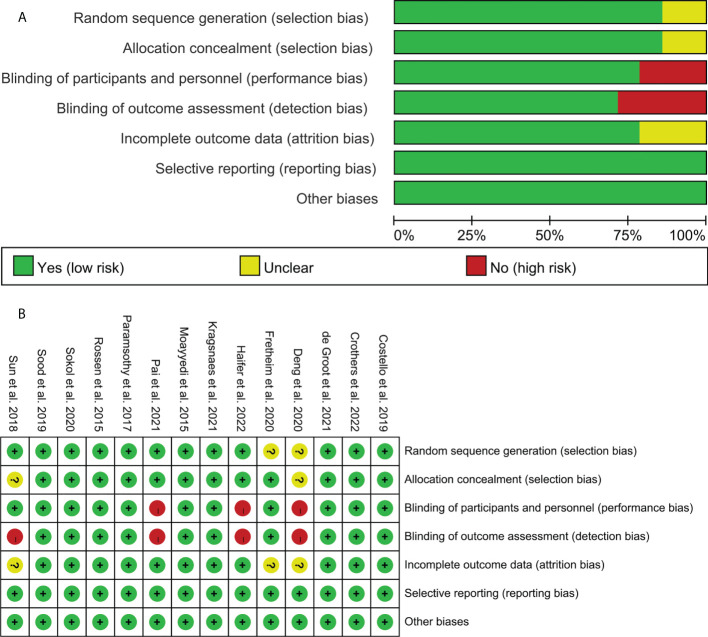
Risk of bias assessment. **(A)** Risk of bias graph. **(B)** Risk of bias summary.

The summary and graph of the risk of bias are shown in **Figure 2**.

#### 3.3.1 Sequence generation

Twelve RCTs described the method of random sequence generation: de Groot et al. (2021) (40), Paramsothy et al. (2017) (42), Rossen et al. (2015) (43), Moayyedi et al. (2015) (44), Costello et al. (2019) (45), Sood et al. (2019) (51), Haifer et al. (2022) (48), Crothers et al. (2022) (45), and Kragsnaes et al. (2021) (53) utilized computer-generated random sequence. Sun et al. (2018) (46) utilized random number table. Sokol et al. (2020) (51) utilized centralized block randomization. Therefore, it is assessed as a low risk of bias. The other RCTs did not describe the method of random sequence generation and were therefore assessed as unclear risk of bias.

Sun et al. (2018) (46) and Deng et al. (2020) (47) did not describe whether to use allocation concealment, so it was rated as unclear risk of bias. The remaining RCTs used describe the available allocation concealment methods. They were considered to have adopted allocation concealment and therefore were assessed as low risk of bias.

#### 3.3.2 Blinding

Sun et al. (2018) (46) only mentioned blinding patients but not blinding data collectors. Therefore, it is assessed as a low risk of bias in “performance bias” and as a high risk of bias in “detection bias”. Deng et al. (2020) (47) did not mention blinding, and the outcome indicators used were subjective (such as clinical remission). Sokol et al. (2020) (50) mentioned the use of single blinding but did not describe the process and objects of blinding. Pai et al. (2021) (52) mentioned the use of blinding for patients and caregivers, but not for members of the research team. Therefore, these three RCTs are assessed as high risk of bias in the blind method. The other RCTs described the blinding of both patients and researchers and were therefore rated as low risk of bias.

#### 3.3.3 Incomplete outcome data and selective reporting

Fretheim et al. (2020) (41), Sun et al. (2018) (46), and Deng et al. (2020) (47) have an incomplete outcome but do not mention the processing method of missing data, so they are assessed as an unclear risk of bias. The intention-to-treat analysis was used in other RCTs, so they were rated as low risk of bias. All RCTs do not have selective reporting and are therefore considered to be a low risk of bias.

#### 3.3.4 Other potential bias

Other sources of bias were not observed in 14 RCTs; therefore, the risks of other biases in the RCTs were low.

### 3.4 Outcomes of type 1 diabetes mellitus

Only one RCT of intestinal microbiota transplantation for the treatment of T1DM has been published [Fretheim et al. (2020) (41)]. The RCT finally included 20 participants (10 in the autologous FMT group and 10 in the allogenic FMT group) for data analysis. For safety, the RCT reported that no serious adverse clinical events nor adverse changes in plasma biochemistry were observed in the two groups.

This RCT showed that compared with the autologous FMT group (control group), the fasting plasma C peptide in the allogenic FMT group at 12 months was lower (348 ± 115 *vs.* 202 ± 85 pmol/L, Student’s t-test p = 0.0049). There was no significant difference between the two groups of blood sugar control (glycated hemoglobin (HbA1c) 46 *vs.* 53.5 mmol/mol, p = 0.19). The RCT also found that the difference between two groups of individual T-cell responses against IA-2, GAD65, and preproinsulin or blood frequencies of islet autoreactive CD8+ T cells (Qdot) was of no statistical significance. There was also no significant difference in the frequency of islet autoreactive CD8+ T cells between the two groups. However, the RCT found that the difference in CD4+ CXCR3+ cells between the treatment group and the control group was statistically significant (p = 0.01) and was negatively correlated with the change in the primary endpoint C-peptide area under the curve (AUC) at 12 months (p = 0.046, rho = −0.47). The RCT observed the changes in the intestinal microbiota in the small intestine after FMT and found that the relative abundance of the microbiota in the control group decreased, while the relative abundance of the treatment group increased. In addition, the study also found that compared with the autologous FMT group (control group), the plasma metabolite of the allogenic FMT group has changed, including 1-myristoyl-2-arachidonoyl-GPC (MA-GPC) (p = 0.02, MWU) and 1-arachidonoyl-GPC (A-GPC) (p = 0.02).

### 3.5 Outcomes of systemic sclerosis

Only one RCT of intestinal microbiota transplantation for the treatment of systemic sclerosis has been published (de Groot et al., 2021). The RCT finally included nine participants (five in the experimental group and four in the placebo group) for data analysis. For safety, there were five adverse events in the experimental group, all of which were mild adverse events, including bloating, constipation, abdominal discomfort (five times), nausea (four times), diarrhea (three times), and vomiting (one time). In the placebo group, a total of four adverse events occurred: one was a serious adverse event, and three were mild adverse events, including diarrhea, nausea (two times), bloating, constipation, vomiting, and fever (one time). This showed that the side effects of anaerobic cultivated human intestinal microbiota (ACHIM) were mild and transient.

This RCT showed that at week 4, compared with one of two placebo controls, three patients in the experimental group reported a major improvement in fecal incontinence. Overall, in four-fifths of the patients in the experimental group (week 4 or 16) and two-quarters of patients in the placebo group (week 4 or 16), improvement in abdominal distension, diarrhea, and/or fecal incontinence was observed. In addition, the RCT also showed that the relative abundance of intestinal microbiota after FMT changed compared with the placebo group [beta diversity (p < 0.02) and number of distinct operational taxonomic units (OTUs) (p < 0.006)]. The genus whose relative abundance increased after the intervention was mainly in Firmicutes, including genera in Ruminococcaceae and Lachnospiraceae. The RCT also recorded modified Rodnan Skin Score (mRSS), new-onset digital ulcers, forced vital capacity (FVC), diffusing capacity of the lungs for carbon monoxide (DLCO), C-reactive protein (CRP), and erythrocyte sedimentation rate (ESR), but the results of the comparison between the groups were not seen.

### 3.6 Outcomes of ulcerative colitis

A total of eight RCTs reported FMT for the treatment of ulcerative colitis, and the outcomes could be pooled, so a meta-analysis was performed.

#### 3.6.1 Clinical remission

**Figure 3:**
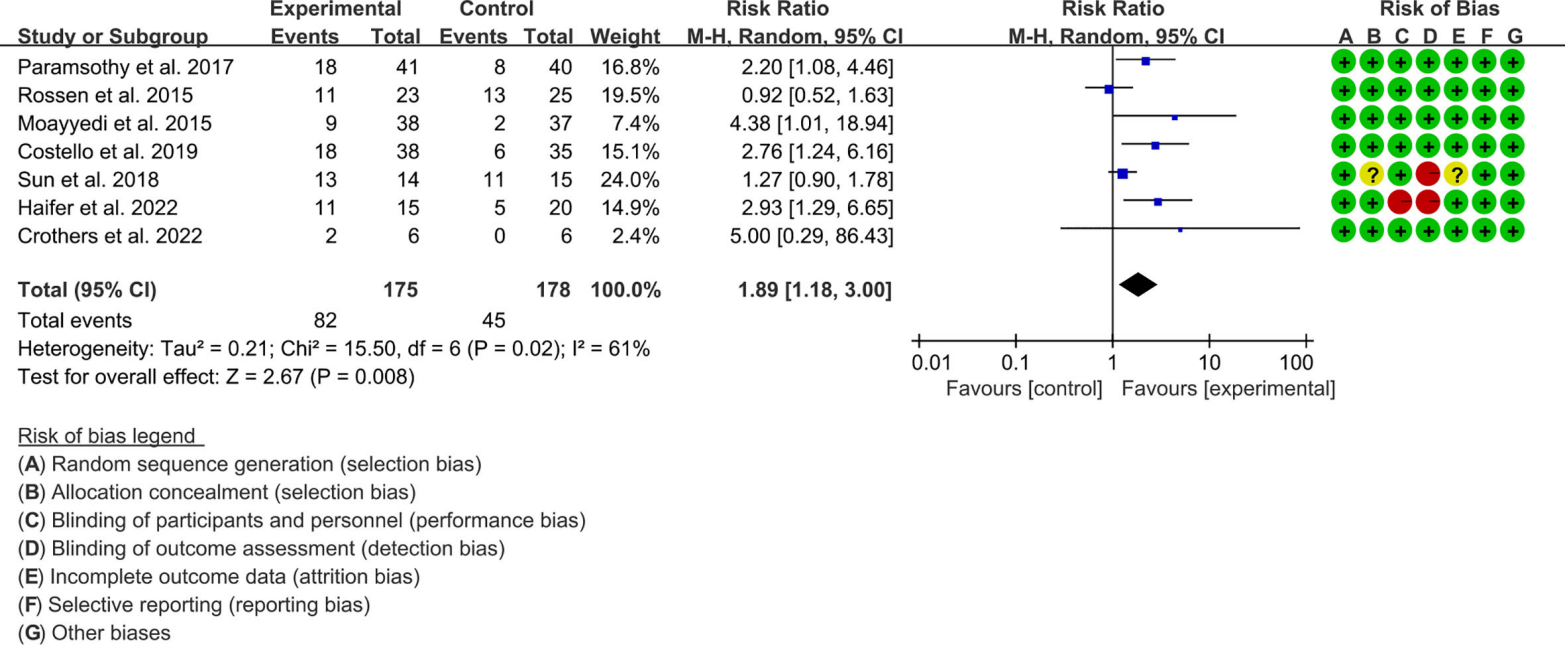
Clinical remission.

Seven RCTs reported clinical remission. The result of the heterogeneity test was I^2^ = 61% and p = 0.02, which indicated that the included RCTs had high heterogeneity, and the random-effects model was used for analysis. The results of the meta-analysis showed that the clinical remission rate of the experimental group was higher than that of the control group [RR 1.89 (1.18, 3.00), p = 0.008, random-effects model] (**Figure 3**).

#### 3.6.2 Clinical response

**Figure 4:**
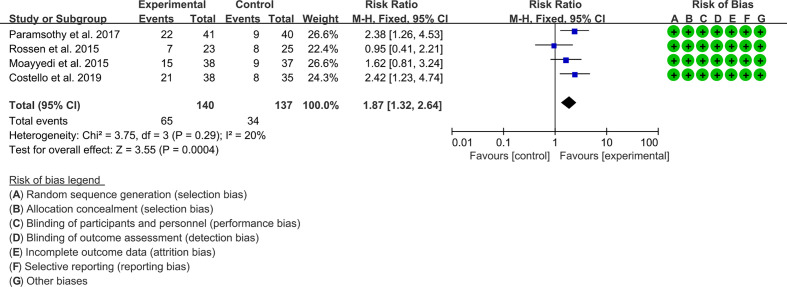
Clinical response.

Four RCTs reported the clinical response. The result of the heterogeneity test was I^2^ = 20% and p = 0.29, which indicated that the included RCTs had low heterogeneity, and the fixed-effects model was used for analysis. The results of the meta-analysis showed that the clinical response rate of the experimental group was higher than that of the control group [RR 1.87 (1.32, 2.64), p = 0.0004, fixed-effects model] (**Figure 4**).

#### 3.6.3 Endoscopic remission

**Figure 5:**
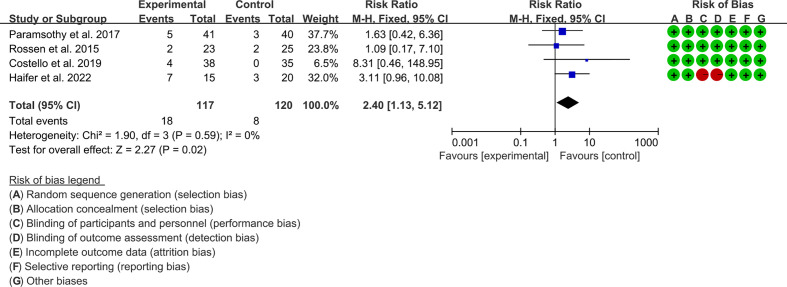
Endoscopic remission.

Four RCTs reported endoscopic remission. The result of the heterogeneity test was I^2^ = 0% and p = 0.59, which indicated that the included RCTs had low heterogeneity, and the fixed-effects model was used for analysis. The results of the meta-analysis showed that the endoscopic remission rate of the experimental group was higher than that of the control group [RR 2.40 (1.13, 5.12), p = 0.02, fixed-effects model] (**Figure 5**).

#### 3.6.4 Endoscopic response

**Figure 6:**
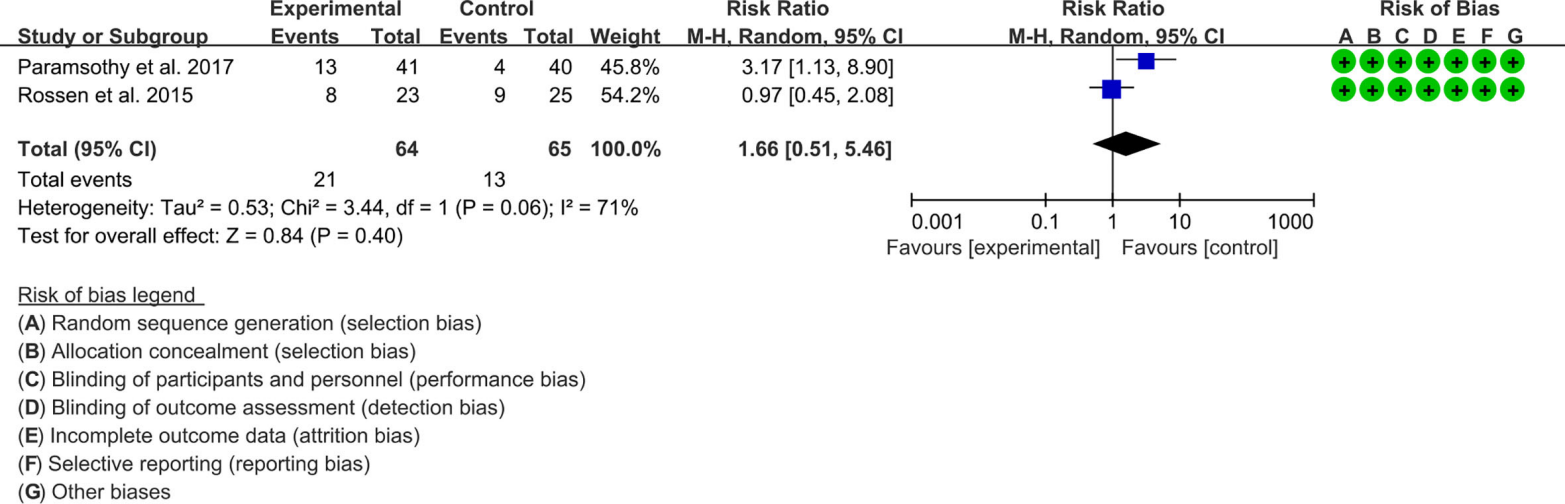
Endoscopic response.

Two RCTs reported the endoscopic response. The result of the heterogeneity test was I^2^ = 71% and p = 0.06, which indicated that the included RCTs had high heterogeneity, and the random-effects model was used for analysis. The results of the meta-analysis showed that the difference between the control and experimental groups was of no statistical significance [RR 1.65 (0.51, 5.48), p = 0.40, random-effects model] (**Figure 6**).

#### 3.6.5 Adverse events

**Figure 7:**
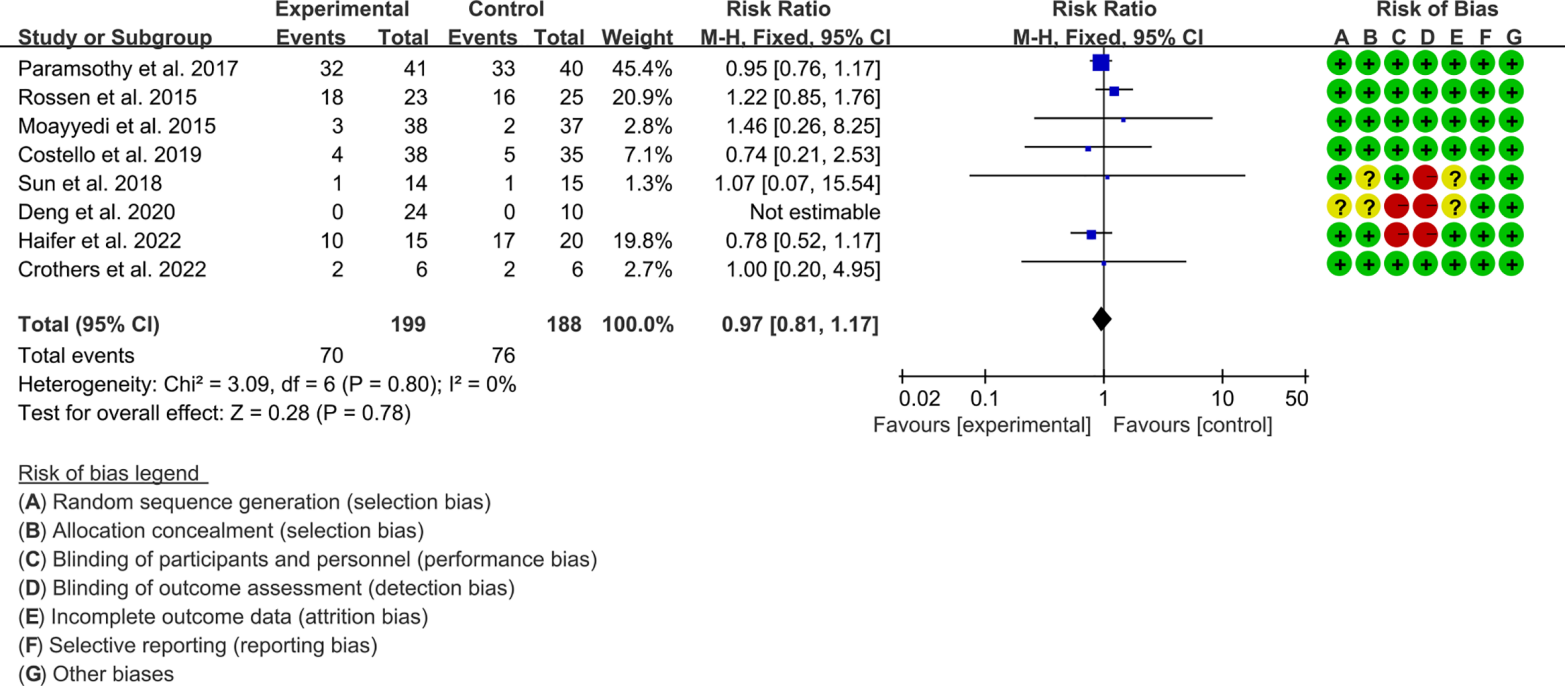
Adverse events.

Eight RCTs reported adverse events. The result of the heterogeneity test was I^2^ = 0% and p = 0.80, which indicated that the included RCTs had low heterogeneity, and the fixed-effects model was used for analysis. The results of the meta-analysis showed that the difference between the control and experimental groups was of no statistical significance [RR 1.02 (0.81, 1.17), p = 0.78, fixed-effects model] (**Figure 7**).

**Figure 8:**
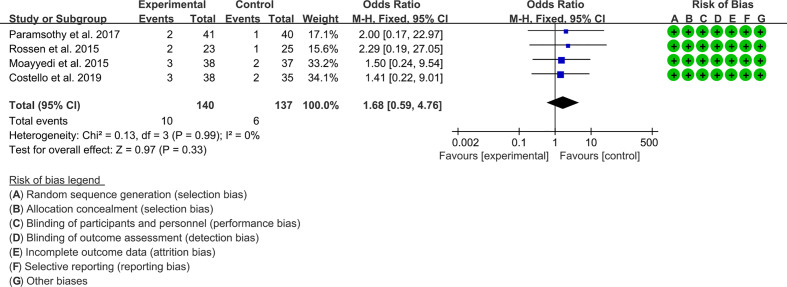
Severe adverse events.

Four RCTs reported severe adverse events. The result of the heterogeneity test was I^2^ = 0% and p = 0.99, which indicated that the included RCTs had low heterogeneity, and the fixed-effects model was used for analysis. The results of the meta-analysis showed that the difference between the control and experimental groups was of no statistical significance [OR 1.68 (0.59, 4.76), p = 0.84, fixed-effects model] (**Figure 8**).

### 3.7 Outcomes of Crohn’s disease

A total of two RCTs reported FMT for the treatment of Crohn’s disease, and the outcomes could be pooled, so a meta-analysis was performed.

#### 3.7.1 Clinical remission

**Figure 9:**
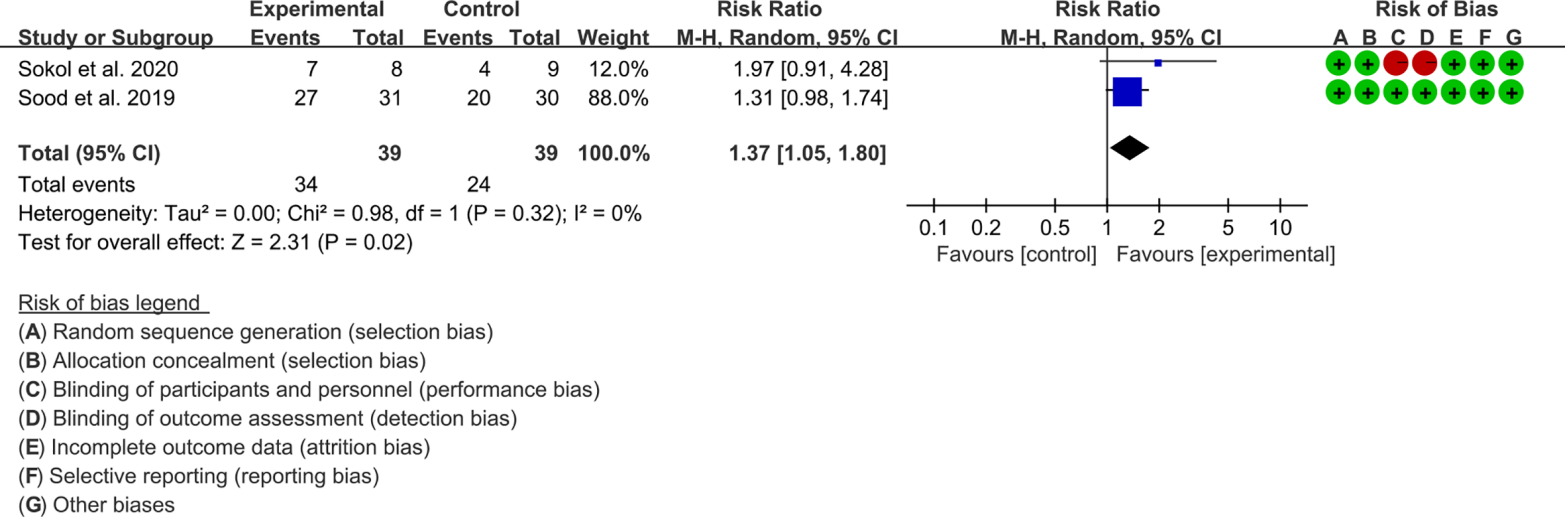
Clinical remission.

Two RCTs reported clinical remission. The result of the heterogeneity test was I^2^ = 65% and p = 0.02, which indicated that the included RCTs had high heterogeneity, and the random-effects model was used for analysis. The results of the meta-analysis showed that the clinical remission rate of the experimental group was higher than that of the control group [RR 1.05 (1.02, 2.79), p = 0.04, random-effects model] (**Figure 9**).

#### 3.7.2 Adverse events

Two RCTs reported clinical remission. Sokol et al. (2020) reported more adverse events but claimed that none of the adverse events were considered to be related to the FMT. Sood et al. (2019) did not report related adverse events (**Figure 9**).

### 3.8 Outcomes of pediatric ulcerative colitis

Only one RCT reported FMT for pediatric ulcerative colitis. The RCT finally included 31 participants (19 in the FMT group and 12 in the placebo enema group) for data analysis. For safety, the RCT reported that no serious adverse clinical events or adverse changes in plasma biochemistry were observed in the two groups. At week 6, 11 children in the FMT group had improvements in UC activity index, C-reactive protein, or fecal calprotectin, as compared with six in the control group (RR 1.8; 95% CI [1.1, 3.7]). At 12 months, nine people in the FMT group maintained a clinical response. Beta diversity increased from baseline to week 6 in the FMT group compared to the placebo group. They found that *Alistipes* spp. and *Escherichia* spp. may be associated with improvements in several clinical outcomes. During the 6-week intervention period, 10 people in the FMT group experienced adverse events compared with five people in the placebo group. Four patients (three in the FMT group and one in the placebo group) had worsening colitis requiring intravenous methylprednisolone. Two FMT patients with a history of *Clostridioides difficile* colitis were diagnosed with *Clostridioides difficile* colitis within 2 weeks of trial withdrawal.

### 3.9 Outcomes of psoriatic arthritis

Only one RCT reported FMT for psoriatic arthritis. The RCT finally included 31 participants (15 in the FMT group and 16 in the placebo enema group) for data analysis. They randomly assigned patients with psoriatic arthritis who were receiving methotrexate to an FMT group and a control group, using a gastroscopically guided approach to transplant gut microbiota or a placebo into the duodenum. No serious adverse events were observed at the 26-week assessment. However, they found a higher rate of treatment failure in the FMT group than in the control group (RR = 3.20; 95% CI [1.06 to 9.62], p = 0.018). The HAQ-DI of the control group was lower than that of the FMT group (p = 0.031). There was no difference in the ratio of ACR20 between the two groups (p > 0.05). However, due to the small number of RCTs, the results need to be interpreted with caution.

### 3.10 Other registered randomized controlled trials related to autoimmune diseases

In addition, after searching in ClinicalTrials.gov and the Chinese Clinical Trials Registry, a total of registered RCTs for FMT treatment of seven types of autoimmune diseases were found: atopic dermatitis, ankylosing spondylitis, lateral sclerosis, rheumatoid arthritis, chronic urticaria, moderate-to-severe chronic plaque psoriasis, and multiple sclerosis (see “**Supplementary Material**—Other registered RCTs related to autoimmune diseases”).

### 3.11 Other registered non-randomized controlled trials related to autoimmune diseases

In addition, after searching in ClinicalTrials.gov and the Chinese Clinical Trials Registry, a total of registered non-RCTs for FMT treatment of five types of autoimmune diseases were found: refractory IgA nephropathy, primary sclerosing cholangitis, multiple sclerosis, T1DM, and gout (see “**Supplementary Material**—Other registered non-RCTs related to autoimmune diseases”).

## 4 Discussion

### 4.1 The mechanism of autoimmune diseases and the intervention mechanism of fecal microbiota transplantation

Autoimmune diseases refer to diseases in which the body’s immune system is abnormally functioning, causing its own tissues to be attacked by the immune system, such as RA and SLE. Its complex pathogenesis has not been fully studied (54, 55). Current studies have shown that the gut microbiota in patients with RA, SLE, spondyloarthritis (SpA), SS, and Behçet’s disease (BD) is significantly different from that in healthy individuals (56–58). Some bacterial species colonized in the human gut, such as *Prevotella copri*, *Ruminococcus gnavus*, and *Lactobacillus salivarius*, are also associated with the pathogenesis of autoimmune diseases (59). The mechanism of intestinal microbiota imbalance in autoimmune diseases may be changing in the metabolic function mediated by intestinal microbiota leading to intestinal microbiota imbalance, which in turn leads to abnormal synthesis or degradation pathways and then to intestinal ecological damage and pathological damage to the body (60, 61). The link between gut microbiota and host immunity suggests that gut microbiota disturbances contribute to the occurrence of autoimmune diseases (62). In addition, current studies have shown that an impaired gut barrier increases the transfer of gut microbes and their constituents, leading to abnormal contact between gut microbes and the host immune system and triggering autoimmunity through various mechanisms (63, 64). For example, studies have shown that *Enterococcus gallinarum* was detected in the liver of patients with systemic lupus erythematosus or autoimmune hepatitis, but not in healthy subjects (65). *E. gallinarum* inoculated in the stomach of lupus-prone mice can cross the intestinal barrier and enter the liver (65). Another study found that some of the bacteria abundant in the gut in patients with autoimmune diseases may originate from the oral cavity (21). Based on the sequence similarity between self-antigens and microbial peptides, immune cells can be cross-activated by microbial peptides to initiate autoimmunity, so molecular mimicry has always been considered an important mechanism involved in the formation of autoimmunity (62). Microorganisms such as *Bacteroides fragilis*, *Candida albicans*, and *Streptococcus sanguis* contain peptides similar to type II collagen, which can cause cross-reactivity in collagen-induced arthritis (66, 67). *Roseburia intestinalis*, *Bacteroides thetaiotaomicron*, etc., can trigger lupus-like symptoms. Furthermore, further studies have shown that dysregulated gut microbiota and their derivatives (e.g., nucleic acids, polysaccharides, metabolites, and toxins) may lead to aberrant activation of innate immune cells leading to inflammation (68, 69). Adaptive lymphocytes also play an important role in autoimmunity, where pathogens or microbial derivatives with pro-inflammatory capabilities can overactivate innate immunity and aberrant antigen presentation, followed by aberrant activation of the adaptive immune system (21). Dendritic cells and macrophages can obtain microbial cells and their derivatives from the intestinal lumen as antigens and further transport these antigens to secondary lymphoid tissues to activate T and B cells. The abnormal activation of the latter two often promotes autoimmune diseases (70–72).

**Figure 10:**
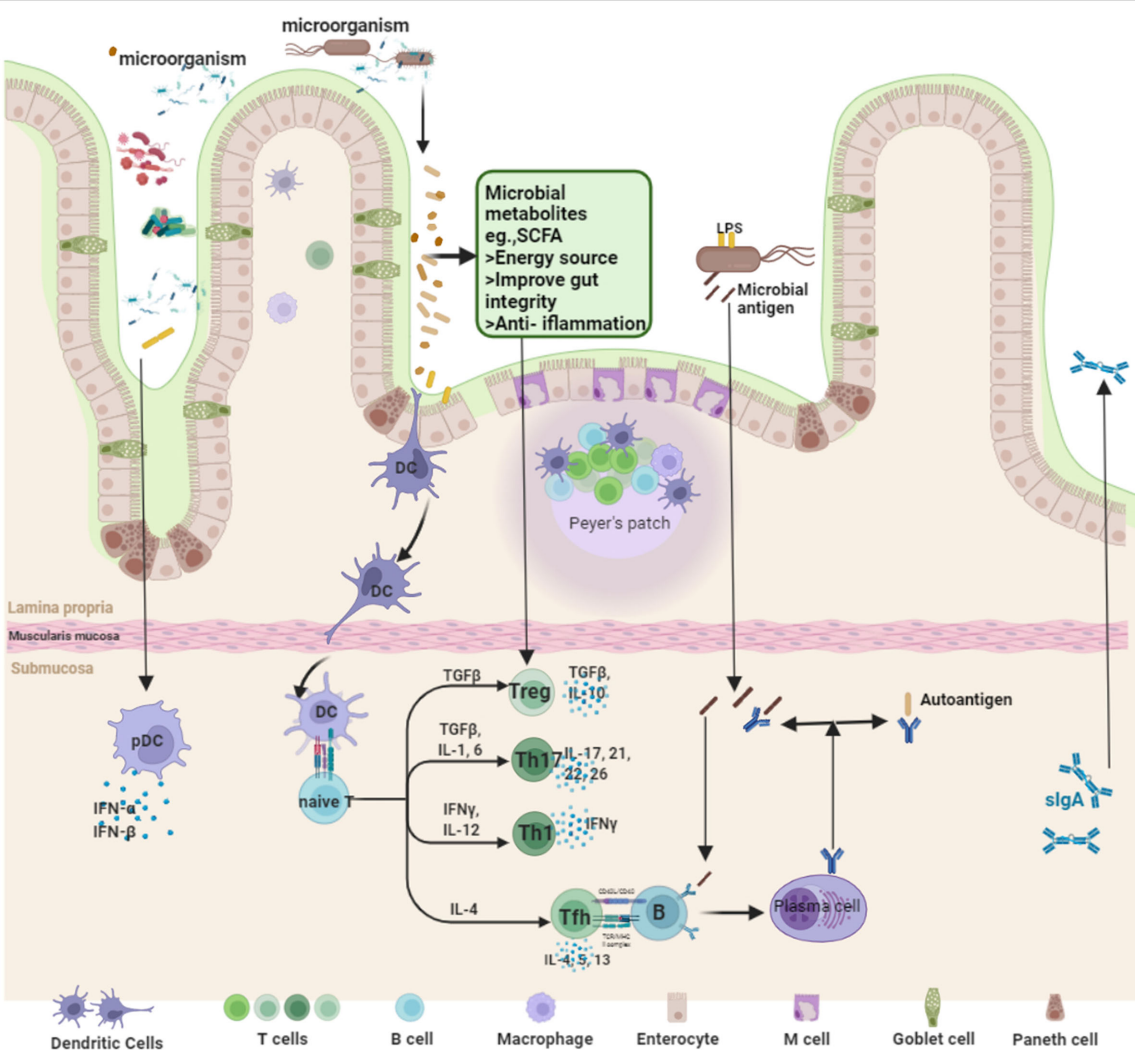
The intervention mechanism of fecal microbiota transplantation.

Improving diet is currently the ideal way to modulate the gut microbiota with few adverse effects, but so far, no exact diet has been found to be beneficial in patients with autoimmune diseases, and strict diet control often results in poor patient compliance (73). Prebiotic and probiotic therapy (colonoscopy fecal microbiota transplantation, oral probiotics, compound microbial preparations, and implantation of beneficial engineered bacteria) modulate gut ecology by competing with harmful microbiota for nutritional and colonizing niches, and in conjunction with specific dietary patterns or prebiotics that support their colonization (74) (**Figure 10**).

### 4.2 Fecal microbiota transplantation for type 1 diabetes mellitus

T1DM is an autoimmune disease caused by the targeted destruction of islet β cells mediated by T lymphocytes. It is mainly a disease caused by mononuclear lymphocytes infiltrating islet beta cells, resulting in massive apoptosis of islet beta cells, decreased insulin secretion, and elevated blood sugar (75, 76). Genetic factors play an important role in the etiology of T1DM. Studies have found many genetic loci associated with T1DM, the most important of which is the human leukocyte antigen (HLA) coding locus, and about 70% of T1DM patients carry HLA alleles (77, 78). However, less than 10% of genetically susceptible individuals develop T1DM (79), but the incidence of T1DM is still increasing year by year, indicating that non-genetic factors play a key role in T1DM. In recent years, the gut microbiota has become a research hotspot as one of the key environmental factors (80–83).

Studies have shown that intestinal grape group imbalance and increased intestinal permeability exist in T1DM patients and animal models. The main manifestations are the decrease in microbiota diversity and probiotic abundance (83, 84), and the regulation of intestinal microbiota through multiple pathways can significantly improve T1DM (85–88). Biassoni et al. (89) used 16S RNA sequencing technology to find that the abundance of Bacteroidetes and Proteobacteria increased in children with T1DM, while the abundance of *Desulfovibrio*, *Biliophilus*, and β-Proteobacteria decreased. Demirci et al. (90) found that the level of Bacteroidetes in the intestinal microbiota of T1DM patients increased, the level of Firmicutes decreased, and the ratio of Firmicutes/Bacteroidetes was significantly reduced. Another clinical study analyzed the gut microbiota of T1DM children and healthy children and found the same results (91). Meanwhile, multiple studies have shown decreased intestinal colonization of *Bifidobacterium* (including *Bifidobacterium adolescentis* and *Bifidobacterium pseudobacteria*) and increased intestinal colonization of *C. albicans* and Enterobacteriaceae (except *Escherichia coli*) in children with T1DM (92–94). Another study also found that children with T1DM had a higher abundance of *Blautia*, and the abundance of *Blautia* genus was positively correlated with HbA1c, the number of T1DM autoantibodies, and tyrosine phosphatase autoantibody (IA-2) titers (95). In addition, the ratio of Bacteroidetes/Firmicutes in the intestinal microbiota of children with T1DM was increased, the abundance of *Faecalibacterium* was negatively correlated with the level of HbA1c, and the abundance of Bacteroidetes was positively correlated with anti-islet autoantibodies (96).

In this systematic review, only one RCT reported FMT for the treatment of T1DM [Fretheim et al. (2020) (40)]. This RCT showed that compared with the autologous FMT group (control group), the fasting plasma C peptide in the allogenic FMT group at 12 months was lower. This RCT also found that the difference in CD4+ CXCR3+ cells between the treatment group and the control group was statistically significant. Nonetheless, due to the small number of RCTs and participants, interpretation of this result remains cautious, and more RCTs are needed to further explore the therapeutic efficacy and safety of FMT in T1DM.

### 4.3 Fecal microbiota transplantation for systemic sclerosis

Systemic sclerosis is an immune-mediated rheumatic disease characterized by skin and internal organ fibrosis and vascular lesions (97). Digestive complications of systemic sclerosis are the second most common complication after skin fibrosis, with an incidence of 90% (98). It is manifested as dysphagia, gastroesophageal reflux, abdominal pain, diarrhea, malnutrition, fecal incontinence, etc., which seriously affect the quality of life and mental health of patients (98, 99). Many studies have confirmed that systemic sclerosis has intestinal microbiota disturbance. Multiple cohort studies have shown significant differences in gut microbiota in patients with systemic sclerosis compared with healthy controls (98–100). For example, the beneficial commensal genera such as probiotics and *Clostridium* were decreased in the gut microbiota of patients with systemic sclerosis, while potentially pathogenic genera, including *Fusarium* and *Ruminococcus*, were increased (101). In addition, a large observational cohort study in Sweden also detected unique microbiota differences in stool samples from 98 patients with systemic sclerosis (102).

In this systematic review, only one RCT reported FMT for the treatment of systemic sclerosis [de Groot et al. (2021) (41)] and showed that at week 4, compared with one of two placebo controls, three patients in the experimental group reported a major improvement in fecal incontinence. Nonetheless, due to the small number of RCTs and participants, interpretation of this result remains cautious, and more RCTs are needed to further explore the therapeutic efficacy and safety of FMT in systemic sclerosis.

### 4.4 Fecal microbiota transplantation for inflammatory bowel disease

Ulcerative colitis, a subtype of inflammatory bowel disease (IBD), is a disease of unknown etiology characterized by chronic, non-specific inflammation of the rectum and colon. The lesions are mainly limited to the intestinal mucosa and submucosa (103, 104). The etiology of ulcerative colitis is still unclear, but it is generally believed that it triggers excessive and inappropriate immune responses in the intestinal lumen mucosa under the combined effects of genetics, environment, and gut microbiota (105). At present, the treatment of ulcerative colitis is mainly based on 5-aminosalicylic acid preparations, corticosteroids, and immunosuppressive drugs. Since these drugs require long-term medication and have large adverse reactions, finding effective new treatment methods is an urgent clinical problem that needs to be solved (106, 107). The recent concept of the pathophysiology of ulcerative colitis further recognizes the important role of the gut microbiota (108). The fecal microbiota test of patients confirmed the existence of obvious intestinal microbiota imbalance, which is characterized by the reduction of intestinal biodiversity and microbial dysfunction, and the changes in intestinal microbiota are closely related to intestinal inflammation (109). The pathological mechanism shows that the disordered intestinal microbiota can damage the intestinal mucosal barrier, change the immune function of the intestinal tract, and secrete a large number of inflammatory mediators, which can lead to the occurrence or aggravation of ulcerative colitis. Some special intestinal microbiota changes are considered to play a key role in the pathogenesis of ulcerative colitis, so it is a new perspective for the current treatment of ulcerative colitis by regulating the structure of patients’ intestinal microbiota to achieve therapeutic effects (110–113). Therefore, measures to correct intestinal dysbiosis have emerged as potential treatments for IBD. The current treatment for altering the intestinal microbiota is mainly oral probiotics of a single species (114, 115). However, the human gut microbiota has more than a thousand different types of microbiota, and the different microbiota often interacts with each other. Therefore, oral probiotics of a single species are often unable to effectively change the intestinal microbiota structure of patients (115). Fecal microbiota transplantation is a treatment method that adjusts the structure of the patient’s intestinal microbiota by transplanting the functional microbiota in the exogenous feces into the patient’s intestine, thereby improving the patient’s intestinal environment. Because fecal microbiota transplantation can directly change the disturbed intestinal microbiota in patients, the use of fecal microbiota transplantation for the treatment of ulcerative colitis has become a research hotspot in recent years (27, 116).

This systematic review and meta-analysis also found that FMT may increase clinical remission, clinical response, and endoscopic remission for patients with ulcerative colitis, and increase clinical remission for patients with Crohn’s disease. These RCTs all reported that FMT did not significantly increase the incidence of adverse reactions, so it can be considered that fecal microbiota transplantation is relatively safe. However, due to the small number of RCTs and participants, interpretation of this result remains cautious; and more RCTs are needed to further explore the therapeutic effect and safety of fecal microbiota transplantation in IBD.

### 4.5 Fecal microbiota transplantation for psoriasis and psoriatic arthritis

Psoriasis affects approximately 2% of the global population and affects all age groups (117). Psoriasis may be related to genetic factors, immune dysfunction, and environmental factors (118, 119). Studies have shown that the pathogenesis of psoriasis is mainly related to the helper T cell (Th)17/IL-23 axis, and the intestinal microbiota can be involved in the differentiation of T cells. For example, segmented filamentous bacteria can induce pro-inflammatory responses in Th17 cells in the gut (120). Experiments have shown that both short-chain fatty acid (SCFA)-producing microbiota and SCFAs can act as potent regulators of T cells in a T cell-mediated inflammatory environment (121–123). Among them, commensal *Clostridium* is the main producer of SCFAs, which can induce the production of IL-10 in the colon, while increasing the number of regulatory T cells (Treg) in the mucosa, and play a key role in intestinal homeostasis (124). *Prevotella*, *Akkermansia muciniphila*, *Faecalibacterium*, and *Ruminococcus* were reduced in both psoriasis (125, 126) and psoriatic arthritis (127). Among them, *Faecalibacterium* and *A. muciniphila* can inhibit Th17 cells and induce the development and expansion of Treg cells, while Treg cells can produce anti-inflammatory cytokines to prevent autoimmunity (128–130). SCFAs can promote the differentiation of lymphoid T cells into Treg cells and induce the expression of IL-10, thereby inhibiting the inflammatory response (131). Meanwhile, SCFAs can also inhibit the activation of lipopolysaccharide-induced chemokine, cytokine, and nuclear factor-κB signaling pathways, relieve the body’s inflammatory response, and improve the symptoms of patients (132). Multiple studies have also demonstrated that psoriatic plaque formation in psoriasis patients is triggered by bacterial DNA in the blood that originates from the intestinal lumen (133, 134). For example, the presence of *Prevotella* and an elevated *Faecalibacterium*/*Bacteroides* ratio can lead to bacterial translocation from the gut to the blood.

In this systematic review, only one RCT reported FMT for psoriatic arthritis. The RCT finally included 31 participants (15 in the FMT group and 16 in the placebo enema group) for data analysis. They randomly assigned patients with psoriatic arthritis who were receiving methotrexate to an FMT group and a control group, using a gastroscopically guided approach to transplant gut microbiota or a placebo into the duodenum. No serious adverse events were observed at the 26-week assessment. However, due to the small number of RCTs, the results need to be interpreted with caution.

### 4.6 Fecal microbiota transplantation for other diseases

There are no related RCTs reported for other diseases, but some of them have been registered in clinical research centers (see “**Supplementary Material**—Other registered non-RCTs related to autoimmune diseases” and “**Supplementary Material**—Other registered RCTs related to autoimmune diseases”). Meanwhile, the current basic research and clinical research have revealed the potential of fecal microbiota transplantation.

RA is a chronic inflammatory autoimmune disease that affects various systems throughout the body, often manifested as joint deformation and morning stiffness, with or without anti-cyclic citrullinated peptide (anti-CCP) antibody positive and rheumatoid factor (RF) abnormalities. Modern medicine believes that the pathogenesis of RA may be related to immune disorders, genetics, and environmental factors, but the specifics are not yet clear. In clinical observations, many scholars have found that there are significant differences in the types and quantities of intestinal microbiota between RA patients and healthy subjects, suggesting a correlation. The researchers analyzed the gut microbiota between 42 untreated RA patients and 10 healthy individuals. They found a fivefold increase in bacilli, a 17-fold increase in lactobacilli, and a significant decrease in *Faecalibacterium* in the untreated RA patient group compared with healthy individuals (135). Metagenome sequencing technology found that untreated RA patients and unrelated healthy controls had similar gut microbiota diversity and richness (22). Several scholars have expounded on the relationship between the intestinal microbiota and the pathogenesis of RA from the perspective of immunology. Wu et al. (136) analyzed the relationship between the intestinal microbiota and the pathogenesis of RA from the molecular level and proposed that the immune response is related to the intestinal mucosal immune system, which is consistent with the view of Catring et al. (137). In addition, in the K/BxN mouse model, the symptoms of RA were alleviated or even disappeared in a sterile state, and the titers of autoantibodies and Th17 cells decreased (138).

SLE is an immune disease that affects multiple organs and spans a large age range. Modern medicine generally believes that the pathogenesis of SLE is related to genetics, hormones, environment, and other factors acting on the body, but the specific mechanism is unknown. A clinical observation detected the stool samples of 30 newly diagnosed SLE patients and 25 healthy people and found that the number of probiotics such as intestinal bifidobacteria and lactobacilli in the SLE group was significantly reduced, and the number of *E. coli* was significantly increased (139). Another study found that the gut Firmicutes/Bacteroidetes (F/B) ratio was significantly lower in SLE patients (140). The study also found that the serum levels of IL-1β, IL-6, IL-17A, IFN-α, and TNF-α in SLE patients were significantly higher than those in the healthy group, while IFN-γ was significantly reduced, which was associated with Firmicutes and Bacteroidetes (141). Several studies have shown that gut microbiota can regulate a variety of inflammatory factors (142–144), and clinical trials have found abnormal concentrations of anti-inflammatory or pro-inflammatory cytokines in the peripheral blood of SLE patients (145, 146). A non-RCT recruited 20 patients with active SLE who still had an SLE Disease Activity Index 2000 (SLEDAI-2K) score of ≥6 despite standard care (147). They found that the SLEDAI-2K score, urine protein/urine creatinine ratio, and serum anti-dsDNA antibody levels decreased significantly after FMT treatment (147). After FMT, the alpha diversity of the gut microbiota of the patients was increased, with significant enrichment of short-chain fatty acid production-related genera, while inflammation-related bacterial taxa decreased, and short-chain fatty acid production in the gut increased. The ratio of CD4+ memory T cells/naive T cells in the peripheral blood of patients and serum IL-6 concentration of SRI-4 responders was significantly decreased (147). This clinical trial confirmed for the first time that FMT, a new medical technology, is safe and effective in the treatment of SLE, providing a new option for the treatment of SLE patients and laying an important foundation for the subsequent promotion and application of larger-scale FMT in the treatment of SLE. It also provides important evidence-based medical evidence for the potential therapeutic value and safety of FMT in autoimmune diseases.

### 4.7 The safety of fecal microbiota transplantation

The currently included RCTs suggest that fecal transplantation is a relatively safe treatment with no increase in adverse events. The most common adverse effects of fecal microbiota transplantation are related to the gastrointestinal tract and include diarrhea, bloating, nausea, vomiting, abdominal pain, and constipation. Other common adverse reactions include fever, dyspnea, headache, and fatigue. These common adverse reactions are generally self-limiting and relieved in the short term. Nevertheless, a few more serious adverse events occurred, mainly due to the aggravation of the above common adverse reactions, as well as *Clostridium difficile* colitis requiring colitis resection, persistent abdominal pain, suspected small bowel perforation, pneumonia, etc. (148, 149). Overall, FMT is a safe therapy, with multiple studies reporting mild and self-limiting side effects.

### 4.8 Factors affecting the efficacy of fecal microbiota transplantation

#### 4.8.1 Selection of donors and disposal of feces

The source of the donor can be classified as follows: 1) according to the source of feces, it can be divided into allogeneic and autologous donors. 2) According to the acquaintance with the patient, the range of donors can be selected from relatives, friends, spouses, and unacquainted volunteers, but there is no clear evidence that these choices are related to the treatment effect. 3) According to the number of donors, it can be divided into single donors and multi-donor. Multi-donor batches have greater microbial diversity than individual donors. Previous studies have shown that donor species richness is a predictor of fecal microbiota transplantation treatment efficacy in patients with intestinal disease. However, multi-donor therapy may limit its ability to compromise beneficial or detrimental donor-specific and microbial content specificity (42). For the selection of allogeneic donors, the existing tests for screening healthy fecal bacteria donors include age, physiology, pathology, psychology, authenticity, time factors, living environment, and donor status (150). However, there are currently no relevant standards for screening donor microbiota. Azimirad et al. (2019) reported two patients with UC and recurrent *Clostridium difficile* infection who experienced adverse events of enterotoxigenic *Clostridium perfringens* infection following fecal microbiota transplantation (151). *C. perfringens* infection may originate from fecal microbiota transplantation, making it necessary to test for this pathogen during donor screening, which may transfer unknown or uncommon pathogens. Therefore, the detection of donor feces also requires stricter control. The disposal of feces after collection also requires a more refined process. The method of manure treatment has gradually shifted from simple mixed filtration to a more refined operation process such as microfiltration and centrifugation. Zhang et al. developed an automated method for the purification of microbial microbiota suspensions with the aim of minimizing the processing time for preserving viable bacteria (150). There was no significant difference in bacterial diversity between the fecal bacteria treated by this method and the original feces. The purification process significantly reduced adverse events but did not alter its efficacy (152).

#### 4.8.2 The path of fecal microbiota transplantation and the frequency of transplantation

The route of administration can be divided into the upper gastrointestinal tract, lower gastrointestinal tract, and swallowing capsules according to the location of injection. The upper gastrointestinal tract transplantation includes a gastroscope, nasogastric tube, and duodenal tube injection, and the lower gastrointestinal tract includes an enema and colonic approach through endoscopic enterotomy (TET). Among these methods, the following digestive transplantation route is more common. Although swallowing the capsules has the advantage of being convenient, due to the large number of capsules that need to be taken at a time, there may not be sufficient doses to maintain long-term treatment. TET has been reported to be a safe and convenient method for the frequency of transplantation and colonic administration (153, 154). Although there are many routes of administration, there is no clear evidence that one route of transplantation is superior to other options. Therefore, in the selection of the transplantation route, factors such as the patient’s psychology, acceptance level, different reactions, and disease degree need to be considered. The frequency of transplantation, compared with single transplantation, multiple treatments can improve the remission rate (155–157), but the specific number of times is still inconclusive.

#### 4.8.3 Pretreatment with antibiotics

Antibiotic pretreatment can increase foreign bacterial colonization in the frequency of transplantation (112). Keshteli et al. (158) conducted a meta-analysis of nine studies (118 patients) and found that the remission rate of patients pretreated with antibiotics before the frequency of transplantation was significantly higher than that of patients not pretreated with antibiotics. Therefore, pretreatment with antibiotics is also a step worth considering when performing the frequency of transplantation.

### 4.9 Strength and limitations and inspiration for future research

The strength is that this is the first systematic review and meta-analysis of FMT in the treatment of autoimmune diseases involving six diseases and 14 RCTs.

The limitations are as follows: 1) the quality of Fretheim et al. (2020) (41), Sun et al. (2018) (46), Deng et al. (2020) (47), Haifer et al. (2022) (48), and Pai et al. (2021) (52) was degraded by the lack of detailed random sequence generation, allocation concealment, and/or blinding information. 2) Although eight diseases are involved, T1DM, systemic sclerosis, pediatric ulcerative colitis, and psoriatic arthritis have only one RCT, Crohn’s disease has only two RCTs, and ulcerative colitis has no more than 10 RCTs; meanwhile, all RCTs involved only 571 participants (less than 1,000). 3) RCTs of FMT intervening in other autoimmune diseases have not been retrieved so far. It may be that the concept of FMT in the treatment of autoimmune diseases has just come out and has received less attention.

Based on the above limitations, it is expected that there will be more large-scale, multi-center RCTs involving multiple participants with high-quality FMT in the treatment of autoimmune diseases to further revise or confirm the conclusions of this systematic review and meta-analysis.

## 5 Conclusion

Based on this systematic review and meta-analysis, the application of FMT in the treatment of autoimmune diseases is effective and relatively safe, and it is expected to be used as a method to induce remission of active autoimmune diseases. The transplantation route, source of fecal bacteria, application of antibiotics, and fecal types had no significant effect on the curative effect. Multiple long-term treatments with FMT could improve the curative effect. However, due to the small number of included RCTs, more high-quality RCTs are needed in the future for further studies to evaluate the long-term safety and efficacy of FMT.

## Data availability statement

The original contributions presented in the study are included in the article/**Supplementary Material**. Further inquiries can be directed to the corresponding authors.

## Author contributions

YD and LZ contributed equally to this work. All authors contributed to the article and approved the submitted version.

## Acknowledgments

The authors thank Xing Li (Department of Foreign Languages, University of Chinese Academy of Sciences, Beijing, China) for assisting with the English translation and writing.

## Conflict of interest

The authors declare that the research was conducted in the absence of any commercial or financial relationships that could be construed as a potential conflict of interest.

## Publisher’s note

All claims expressed in this article are solely those of the authors and do not necessarily represent those of their affiliated organizations, or those of the publisher, the editors and the reviewers. Any product that may be evaluated in this article, or claim that may be made by its manufacturer, is not guaranteed or endorsed by the publisher.
