# Supplementary material for: Safety and efficacy of fecal microbiota transplantation for autoimmune diseases and autoinflammatory diseases: A systematic review and meta-analysis
Source: Front Immunol. 2022 Sep 30;13:944387. doi: 10.3389/fimmu.2022.944387 (PMC9562921; doi:10.3389/fimmu.2022.944387)
Supplement: Supplementary file 1 [file Table_1.docx]

**Table S1.** Search Strategies for Pubmed and Embase

| **PubMed** | (Fecal Microbiota Transplantations OR Microbiota Transplantation, Fecal OR Microbiota Transplantations, Fecal OR Transplantation, Fecal Microbiota OR Transplantations, Fecal Microbiota OR Fecal Microbiota Transplant OR Fecal Microbiota Transplants OR Microbiota Transplant, Fecal OR Microbiota Transplants, Fecal OR Transplant, Fecal Microbiota OR Transplants, Fecal Microbiota OR Fecal Microbiome Transplantation OR Fecal Microbiome Transplantations OR Microbiome Transplantation, Fecal OR Microbiome Transplantations, Fecal OR Transplantation, Fecal Microbiome OR Transplantations, Fecal Microbiome OR Fecal Transplant OR Fecal Transplants OR Transplant, Fecal OR Transplants, Fecal OR Donor Feces Infusion OR Donor Feces Infusions OR Feces Infusion, Donor OR Feces Infusions, Donor OR Infusion, Donor Feces OR Infusions, Donor Feces OR Intestinal Microbiome Transplant OR Intestinal Microbiome Transplants OR Microbiome Transplant, Intestinal OR Microbiome Transplants, Intestinal OR Transplant, Intestinal Microbiome OR Transplants, Intestinal Microbiome OR Intestinal Microbiota Transfer OR Intestinal Microbiota Transfers OR Microbiota Transfer, Intestinal OR Microbiota Transfers, Intestinal OR Transfer, Intestinal Microbiota OR Transfers, Intestinal Microbiota OR Intestinal Microbiota Transplantation OR Intestinal Microbiota Transplantations OR Microbiota Transplantation, Intestinal OR Microbiota Transplantations, Intestinal OR Transplantation, Intestinal Microbiota OR Transplantations, Intestinal Microbiota OR Intestinal Microbiome Transplantation OR Intestinal Microbiome Transplantations OR Microbiome Transplantation, Intestinal OR Microbiome Transplantations, Intestinal OR Transplantation, Intestinal Microbiome OR Transplantations, Intestinal Microbiome OR Intestinal Microbiota Transplant OR Intestinal Microbiota Transplants OR Microbiota Transplant, Intestinal OR Microbiota Transplants, Intestinal OR Transplant, Intestinal Microbiota OR Transplants, Intestinal Microbiota OR Intestinal Microbiome Transfer OR Intestinal Microbiome Transfers OR Microbiome Transfer, Intestinal OR Microbiome Transfers, Intestinal OR Transfer, Intestinal Microbiome OR Transfers, Intestinal Microbiome OR Fecal Microbiota Transfer OR Fecal Microbiota Transfers OR Microbiota Transfer, Fecal OR Microbiota Transfers, Fecal OR Transfer, Fecal Microbiota OR Transfers, Fecal Microbiota OR Fecal Transplantation OR Fecal Transplantations OR Transplantation, Fecal OR Transplantations, Fecal)  AND  ((Autoimmune diseases OR Autoantibodies OR Autoimmunity OR Multiple Sclerosis OR Multiple Sclerosis, Chronic Progressive OR Multiple Sclerosis, Relapsing-Remitting OR Narcolepsy OR Hypersomnolence, Idiopathic OR Addison's Disease OR Diabetes Mellitus, Type 1 OR Graves Disease OR Hashimoto Disease OR Hepatitis, Autoimmune OR Coeliac Disease OR Crohn Disease OR Anemia, Pernicious OR Liver Cirrhosis, Biliary OR Colitis, Ulcerative OR Antiphospholipid Syndrome OR Purpura, Thrombocytopenic, Idiopathic OR Arthritis, Rheumatoid OR Mucocutaneous Lymph Node Syndrome OR Rheumatic Fever OR Giant Cell Arteritis OR Alopecia Areata OR Dermatitis Herpetiformis OR Vitiligo OR Scleroderma, Systemic OR Scleroderma, Localized OR Scleroderma, Diffuse OR Scleroderma, Limited OR Sjögren's syndrome OR Lupus Erythematosus, Systemic))  AND  (random* controlled trial [pt] OR controlled clinical trial* [pt] OR randomized [tiab] OR placebo [tiab] OR drug therapy [sh] OR random* [tiab] OR trial* [tiab] OR group* [tiab])  NOT  (animals [mh] NOT humans [mh]) |
| --- | --- |
| **EMBASE** | 1 Autoimmune diseases/  2 Autoantibodies/  3 Autoimmunity/  4 Multiple Sclerosis/  5 Multiple Sclerosis, Chronic Progressive/  6 Multiple Sclerosis, Relapsing-Remitting/  7 Narcolepsy/  8 Hypersomnolence, Idiopathic/  9 Addison's Disease/  10 Diabetes Mellitus, Type 1/  11 Graves Disease/  12 Hashimoto Disease/  13 Hepatitis, Autoimmune/  14 Coeliac Disease/  15 Crohn Disease/  16 Anemia, Pernicious/  17 Liver Cirrhosis, Biliary/  18 Colitis, Ulcerative/  19 Antiphospholipid Syndrome/  20 Purpura, Thrombocytopenic, Idiopathic/  21 Arthritis, Rheumatoid/  22 Mucocutaneous Lymph Node Syndrome/  23 Rheumatic Fever/  24 Giant Cell Arteritis/  25 Alopecia Areata/  26 Dermatitis Herpetiformis/  27 Vitiligo/  28 Scleroderma, Systemic/  29 Scleroderma, Localized/  30 Scleroderma, Diffuse/  31 Scleroderma, Limited/  32 Sjögren's syndrome/  33 Lupus Erythematosus, Systemic/  34 1-33/or  35 Fecal Microbiota Transplantations/  36 Microbiota Transplantation, Fecal/  37 Microbiota Transplantations, Fecal/  38 Transplantation, Fecal Microbiota/  39 Transplantations, Fecal Microbiota/  40 Fecal Microbiota Transplant/  41 Fecal Microbiota Transplants/  42 Microbiota Transplant, Fecal/  43 Microbiota Transplants, Fecal/  44 Transplant, Fecal Microbiota/  45 Transplants, Fecal Microbiota/  46 Fecal Microbiome Transplantation/  47 Fecal Microbiome Transplantations/  48 Microbiome Transplantation, Fecal/  49 Microbiome Transplantations, Fecal/  50 Transplantation, Fecal Microbiome/  51 Transplantations, Fecal Microbiome/  52 Fecal Transplant/  53 Fecal Transplants/  54 Transplant, Fecal/  55 Transplants, Fecal/  56 Donor Feces Infusion/  57 Donor Feces Infusions/  58 Feces Infusion, Donor/  59 Feces Infusions, Donor/  60 Infusion, Donor Feces/  61 Infusions, Donor Feces/  62 Intestinal Microbiome Transplant/  63 Intestinal Microbiome Transplants/  64 Microbiome Transplant, Intestinal/  65 Microbiome Transplants, Intestinal/  66 Transplant, Intestinal Microbiome/  67 Transplants, Intestinal Microbiome/  68 Intestinal Microbiota Transfer/  69 Intestinal Microbiota Transfers/  70 Microbiota Transfer, Intestinal/  71 Microbiota Transfers, Intestinal/  72 Transfer, Intestinal Microbiota/  73 Transfers, Intestinal Microbiota/  74 Intestinal Microbiota Transplantation/  75 Intestinal Microbiota Transplantations/  76 Microbiota Transplantation, Intestinal/  77 Microbiota Transplantations, Intestinal/  78 Transplantation, Intestinal Microbiota/  79 Transplantations, Intestinal Microbiota/  80 Intestinal Microbiome Transplantation/  81 Intestinal Microbiome Transplantations/  82 Microbiome Transplantation, Intestinal/  83 Microbiome Transplantations, Intestinal/  84 Transplantation, Intestinal Microbiome/  85 Transplantations, Intestinal Microbiome/  86 Intestinal Microbiota Transplant/  87 Intestinal Microbiota Transplants/  88 Microbiota Transplant, Intestinal/  89 Microbiota Transplants, Intestinal/  90 Transplant, Intestinal Microbiota/  91 Transplants, Intestinal Microbiota/  92 Intestinal Microbiome Transfer/  93 Intestinal Microbiome Transfers/  94 Microbiome Transfer, Intestinal/  95 Microbiome Transfers, Intestinal/  96 Transfer, Intestinal Microbiome/  97 Transfers, Intestinal Microbiome/  98 Fecal Microbiota Transfer/  99 Fecal Microbiota Transfers/  100 Microbiota Transfer, Fecal/  101 Microbiota Transfers, Fecal/  102 Transfer, Fecal Microbiota/  103 Transfers, Fecal Microbiota/  104 Fecal Transplantation/  105 Fecal Transplantations/  106 Transplantation, Fecal/  107 Transplantations, Fecal/  108 35-107/or  109 crossover procedure/  110 double blind procedure/  111 single blind procedure/  112 triple blind procedure/  113 randomized controlled trial/  114 109-113/or  115 34 and 108  116 114 and 115 |
