# Supplementary material for: Safety and efficacy of fecal microbiota transplantation for autoimmune diseases and autoinflammatory diseases: A systematic review and meta-analysis
Source: Front Immunol. 2022 Sep 30;13:944387. doi: 10.3389/fimmu.2022.944387 (PMC9562921; doi:10.3389/fimmu.2022.944387)
Supplement: Supplementary file 3 [file Table_3.docx]

**3.10 Other registered RCTs related to autoimmune diseases**

In addition, after searching in ClinicalTrials.gov and the Chinese Clinical Trials Registry, a total of registered RCTs for FMT treatment of 7 types of autoimmune diseases were found: Atopic dermatitis, ankylosing spondylitis, lateral sclerosis, rheumatoid arthritis, chronic urticaria, moderate to severe chronic plaque psoriasis, multiple sclerosis (Table S1-S7)

**3.10.1 Atopic dermatitis**

The registered RCT of Atopic dermatitis were shown in Table S1.

Table S1 Atopic dermatitis (NCT04283968)

| Trial name or title | Efficacy of Fecal Microbial Transplantation Treatment in Adults With Atopic Dermatitis |
| --- | --- |
| Methods | Interventional (Clinical Trial), randomized |
| Participants | Inclusion Criteria: Patients who are ≥18 years of age, with moderate-to-severe atopic dermatitis, as defined by a Scoring Atopic Dermatitis Score (SCORAD) score ≥25, with disease duration minimum of 3 years that was inadequately controlled by topical and systemic therapy Exclusion Criteria: 1. Age under 18 years 2. Pregnancy 3. Another concomitant active dermatologic disease. 4. Receiving systemic therapy including phototherapy within 4 weeks before the beginning of the study. 5. receiving any antibiotic or probiotic treatment within 2 weeks before the beginning of the study or during the study |
| Interventions | Intervention: Fecal Microbial Transplantation Comparison: placebo fecal transplantation |
| Outcomes | Primary Outcome Measures: 1. Assessment of the change in the severity of Atopic Dermatitis after treatment with Fecal Microbial Transplantation Secondary Outcome Measures: 1.Association between the improvement in disease severity and the degree of bacterial strain transmission from donor to patient. Other Outcome Measures: 1. Assessment of the change in the IgE level after treatment with Fecal microbial transplantation |
| Starting date | October 15, 2020 |
| Contact information | Jacob Mashiah, MD, Tel Aviv Sourasky medical center Tel Aviv, Israel, 64239 |
| Notes | Enrolling by invitation; Estimated Enrollment: 50 |

**3.10.2 Ankylosing spondylitis**

The registered RCT of Ankylosing spondylitis were shown in Table S2.

Table S2 ankylosing spondylitis (NCT03726645)

| Trial name or title | The Effect of Fecal Microbiota Transplantation in Ankylosing Spondylitis (AS) Patients |
| --- | --- |
| Methods | Interventional (Clinical Trial), randomized |
| Participants | Inclusion Criteria: 1. Diagnosis of AS by either the 1984 New York criteria or the ASAS (Assessment of SpondyloArthritis International Society) criteria for axial spondyloarthritis. 2. Active disease measured by BASDAI > 4. 3. Availability of consecutive fecal samples over 1 year period. 4. Compliance to attend ileocolonoscopy and FMT procedure. Exclusion Criteria: 1. Diagnosis of inflammatory bowel disease. 2. Antibiotic therapy within the last 3 months. 3. Use of any probiotics within the last 3 months. 4. Pregnancy. 5. Unability to provide a written consent. 6. Other reason which by the opinion of the investigator makes patient ineligible for the study. |
| Interventions | Intervention: Allogeneic fecal microbiota transplantation (from donor) Comparison: Autologous fecal microbiota transplantation (own stool) |
| Outcomes | Primary Outcome Measures  : 1. The effect of FMT (fecal microbiota transplantation) on the clinical activity of ankylosing spondylitis (AS) as assessed by change in BASDAI (Bath Ankylosing Spondylitis Disease Activity Index).  Secondary Outcome Measures  : 1. The effect of FMT on the clinical activity of AS as assessed by change in BASFI (Bath Ankylosing Spondylitis Functional Index).  2. The effect of FMT on the clinical activity of AS as assessed by change in MASES (Maastricht Ankylosing Spondylitis Enthesitis Score).  3. The effect of FMT on C-reactive protein (CRP) concentration. 4. The effect of FMT on erythrocyte sedimentation rate (ESR) level.  5. The effect of FMT on gut wall inflammation as assessed by change in fecal calprotectin (F-calpro) level. 6. The effect of FMT on gut microbiota composition in AS patients. 7. Association between specific intestinal pathogens and disease activity as assessed by BASDAI score. 8. Association between specific intestinal pathogens and disease activity as assessed by CRP concentration. 9. Association between gut wall cytokine expression and disease activity as assessed by BASDAI score.  10. Association between gut wall inflammasome activity and disease activity as assessed by BASDAI score. 11. Association between gut wall cytokine expression and disease activity as assessed by CRP concentration. 12. Association between gut wall inflammasome activity and disease activity as assessed by CRP concentration. 13. Association between F-Calpro level and disease activity as assessed by BASDAI score. 14. Association between F-Calpro level and disease activity as assessed by CRP concentration.  15. The effect of FMT on gut wall permeability as assessed by blood zonulin concentration.  16. The effect of FMT on gut wall bacterial penetrance as assessed by lipopolysaccharide (LPS) concentration. 17. The effect of FMT on gastrointestinal symptoms as assessed by GSRS (The Gastrointestinal Symptom Rating Scale). |
| Starting date | October 24, 2018 |
| Contact information | Kari K Eklund, PhD, MD Hospital District of Helsinki and Uusimaa, Department of Rheumatology Helsinki, Uusimaa, Finland, 00029 |
| Notes | Enrolling by invitation; Actual Enrollment: 20 |

**3.10.3 Lateral sclerosis**

The registered RCT of lateral sclerosis were shown in Table S3.

Table S3 lateral sclerosis (NCT03766321)

| Trial name or title | Fecal Microbiota Transplantation Effect on Amyotrophic Lateral Sclerosis Patients (FETR-ALS) |
| --- | --- |
| Methods | Interventional (Clinical Trial), randomized |
| Participants | Inclusion Criteria: Patients diagnosed with a laboratory supported, clinically "probable" or "definite" amyotrophic lateral sclerosis according to the Revised El Escorial criteria (Brooks, 2000) Sporadic or familial ALS Female or male patients aged between 18 and 70 years old Disease duration from symptoms onset no longer than 18 months at the screening visit Patients treated with a stable dose of Riluzole (100 mg/day) for at least 30 days prior to screening Patients with a weight > 50 kg and a BMI ≥18 Patients with a FVC (Forced Vital Capacity) equal or more than 70% predicted normal value for gender, height, and age at the screening visit Patients able and willing to comply with study procedures as per protocol Patients able to understand, and capable of providing informed consent at screening visit prior to any protocol-specific procedures Use of effective contraception both for males and females Exclusion Criteria: Known organic gastrointestinal disease History of gastrointestinal malignancy; ongoing malignancies Use of immunosuppressive or chemotherapy within the past 2 years Celiac disease and/or food (e.g.lactose) intolerance Previous gastrointestinal surgery Any condition that would make endoscopic procedures contraindicated Acute infections requiring antibiotics Antimicrobial treatment or probiotics 4 weeks prior to screening Severe comorbidities (heart, renal, liver failure); severe renal (eGFR< 30ml/min/1.73m2), or liver failure or liver aminotransferase (ALT/AST > 2x Upper limit of normal), Autoimmune diseases, inflammatory disorders (SLE, Rheumatoid arthritis, connective tissue disorder) or chronic infections (HIV, hepatitis B or C infection) Abuse of alcohol or drugs HIV, tuberculosis, hepatitis Participation in clinical trials <30 days before screening Existing blood dyscrasia (e.g., myelodysplasia) White blood cells<4,000/mm³, platelets count<100,000/mm³, hematocrit<30% Patients who underwent non-invasive ventilation, tracheotomy and /or gastrostomy Women who are pregnant or breastfeeding |
| Interventions | Intervention: FMT (Fecal microbiota transplantation will be performed during two endoscopic procedures (baseline and at 6 months) by allogenic infusion of collected feces in the duodenum-jejunum.) Comparison: Placebo (patients will undergo endoscopic procedure with biopsy during sedation but without any kind of intervention) |
| Outcomes | Primary Outcome Measures: 1. Change in Tregs number Secondary Outcome Measures: 1. Change in T cell subsets frequency in blood and gut tissue samples 2. Change in heavy neurofilaments levels in CSF 3. Changes in levels of pro-inflammatory cytokines and cytokines linked to T cell proliferation and differentiation 4. Changes in microbiota profile 5. Incidence of Adverse Events 6. Tracheostomy free survival 7. Forced vital capacity (FVC) 8. disease progression 9. quality of life: Amyotrophic Lateral Sclerosis Assessment Questionnaire (ALSAQ-40) 10. Number of Adverse Events 11. Number of Patients with Adverse Events |
| Starting date | July 1, 2020 |
| Contact information | Jessica Mandrioli, MD, Azienda Ospedaliero Universitaria di Modena, Modena, Italy |
| Notes | Recruiting; Estimated Enrollment: 42 |

**3.10.4 Rheumatoid arthritis**

The registered RCT of Rheumatoid arthritis were shown in Table S4.

Table S4 rheumatoid arthritis (NCT03944096)

| Trial name or title | Efficacy and Safety of Fecal Microbiota Transplantation in Patients With Rheumatoid Arthritis Refractory to Methotrexate (FARM) |
| --- | --- |
| Methods | Interventional (Clinical Trial), randomized |
| Participants | Inclusion Criteria: Age 18-65 years with informed consent Fulfill the 2010 ACR/EULAR classification criteria for rheumatoid arthritis Positive RF or anti-CCP antibody on screening Have active RA shown by swollen joint count(SJC)≥4 and tender joint count(TJC)≥4 and ESR >28 mm/hr or C-reactive protein > 1.5 ULN Have received methotrexate for 3 months or longer and at a stable dose of 7.5 to 25 mg/week (extremes included) for at least four weeks prior to screening and willing to continue on this regimen for the duration of the study. Class I, II or III of the ACR 1991 Revised Criteria for Global Functional Status in RA If taking oral steroids, these should be at a dose ≤10 mg/day of prednisone or prednisone equivalent and stable for at least four weeks prior to screening; If taking non-steroidal anti-inflammatory drugs (NSAIDs), these must be at a stable dose for at least two weeks prior to screening; Female subjects must have a negative pregnancy test unless they are surgically sterile or have been post-menopausal for at least one year (12 consecutive months without menses); Women of childbearing potential must use a medically acceptable means of birth control and agree to continue its use during the study and for at least four weeks after the last dose of study drug. Sexually active men must agree to use a medically acceptable form of contraception during the study and continue its use for at least 3 months after the last dose of study drug; and Willing to suspend the use of other adjuvant treatment for the duration of the study including acupuncture, massage, etc. Exclusion Criteria: Pregnant, lactating or further fertility requirements History of any inflammatory rheumatological disorders other than RA; Previously received any biologic agents. Treatment with disease-modifying antirheumatic drugs (DMARDs), other than background methotrexate; Receipt of an intra-articular or parenteral corticosteroid injection within four weeks prior to screening; Active or chronic infection, including HIV, HCV, HBV, tuberculosis. Malignancy or history of malignancy. Severe, progressive, or uncontrolled cardiac, pulmonary, renal, hepatic, gastrointestinal, hematologic, metabolic, endocrine or neurologic disease unable to undergo colonoscopy. |
| Interventions | Intervention: Allogeneic fecal microbiota transplantation (from donor) Comparison: Autologous fecal microbiota transplantation (own stool) |
| Outcomes | Primary Outcome Measures  : 1. The American College of Rheumatology 20 (ACR20) response at 16 weeks Secondary Outcome Measures  : 1. The American College of Rheumatology 50/70 (ACR50/ACR70) response at 16 weeks  2. The American College of Rheumatology 20/50/70 (ACR20/ACR50/ACR70) response at 24 weeks  3. The Disease Activity Score-28 (DAS28) response at 16 weeks 4. The Disease Activity Score-28 (DAS28) response at 24 weeks  5. The European League Against Rheumatism (EULAR) response at 16 weeks 6. Health Assessment Questionnaire without Didability Index (HAQ-DI) at 16 weeks 7. Incidence of adverse events and sever adverse events (SAE) during the study |
| Starting date | April 30, 2019 |
| Contact information | Yue Li, MD, Peking Union Medical College Hospital, Beijing, China, 100032 |
| Notes | Recruiting; Estimated Enrollment: 30 |

**3.10.5 Chronic urticaria**

The registered RCT of chronic urticaria were shown in Table S5.

Table S5 chronic urticaria (ChiCTR1900021307)

| Trial name or title | Preliminary study for the treatment of chronic urticaria with fecal microbiota transplantation: a randomized controlled trial |
| --- | --- |
| Methods | Interventional (Clinical Trial), randomized |
| Participants | Inclusion criteria: 1. Volunteer to participate in the study, comply with the study requirements, sign the informed consent form, and have the intention and ability to complete the schedule record for more than 80% of the study period; 2. Males or females, aged 18 to 65 years; 3. More than 6 months after the diagnosis of chronic urticaria, patients still had at least 2 episodes of anemone and pruritus per week after taking the recommended dose of H1 receptor antihistamine regularly for 6 weeks before inclusion; 4. Pruritus VAS<7 points; 5. Accompanied by chronic constipation or chronic diarrhea and other gastrointestinal symptoms for more than 3 months.  Exclusion criteria: 1. Induced chronic urticaria, such as: artificial urticaria, cold sex urticaria, delay pressure sex urticaria, hot sex urticaria, solar sex urticaria, vibration sex urticaria and angioedema, cholinergic urticaria, water sex urticaria, sex urticaria, exercise induced urticaria with clear incentives; 2. Other diseases associated with urticaria or vascular edema, such as urticaria vasculitis, polymorphic erythema, mast cell hyperplasia, hereditary or acquired angioedema, lymphoma, leukemia and generalized tumors; 3. Within 7-14 days before enrollment, the uas7-day total score (UAS7) >20 points, or the total score of itch symptoms <2 points or >14 points, or the itch score/ventilator score in one day or more≥3; 4. Patients who are in the active stage of atopic dermatitis, bullous pemphigoid, herpetic dermatitis, pruritus senile or other skin diseases with obvious itching, or who have a previous medical history of the above diseases; 5. Those who were active in infectious diseases (except colds) within 2 weeks before enrollment; Previous history of chronic or recurrent infectious diseases; 6. Those who have received immunosuppressive agents, biological agents and systemic glucocorticoid treatment within 30 days before enrollment; For those who use the following drugs for continuously within 30 days or at least 5 days every other day: topical corticosteroids (prescription or over prescription), hydroxychloroquine, methotrexate, cyclosporine, cyclophosphamide and other immunomodulators; Patients who have received intravenous injection of immunoglobulin or plasma exchange within 30 days; 7. Patients who underwent surgery for other diseases or may be required to undergo surgery within 2 months before enrollment; Those who have received or are about to receive live vaccines within 6 weeks; Used antimicrobial drugs, probiotics, large doses of vitamins within 30 days, and received treatment to regulate intestinal microecology; If you are using other experimental drugs at the time of enrollment or within 5 times half-life of the experimental drugs or within 30 days (whichever is the older); 8. Previous allergic history of loratadine or local anesthetic drugs; A history of anaphylactic shock; 9. Pregnancy or recent pregnancy preparation is possible; Women who are likely to have children (defined as all women who are physically able to become pregnant, unless an effective contraceptive method is used throughout the study period (specific agreement is made in the protocol on the possibility of having children and the effective contraceptive method); Pregnant or lactating women (pregnancy is defined as the state of a woman after conception and before termination of pregnancy, subject to positive results of hCG laboratory tests). 10. Important viscera serious problems: including but not limited to cardiovascular (uncontrolled hypertension, systolic blood pressure, 160 MMHG or 95 MMHG or higher and /or diastolic blood pressure, heart failure: New York heart association class III/IV), nervous system, liver, kidney (serum creatinine level > 2.0 mg/dl (176.8 u mol/L), the blood system (into a group of white blood cells (WBC) count when < 2500 / mu L, or platelets < 100000 / mu L or neutrophils < 1500 / mu L or hemoglobin < 8.5 g/dl) disease; Previous history of lymphoproliferative disease; Patients with a history of malignancy within the first 5 years of admission (except for Bowen's disease, basal cell carcinoma, actinic keratosis and resected carcinoma in situ or non-invasive malignant colonic polyps that have been treated and have no evidence of recurrence within 12 weeks);Congenital immunodeficiency or low immunity. 11. Inability to complete treatment and follow up for any reason. |
| Interventions | Intervention: FMT+ external calamine lotion + external medical emollient Comparison: Gastrointestinal endoscopy and normal saline injection + external calamine lotion + external medical emollient |
| Outcomes | Outcomes:  Structure and diversity of faeces, intestinal mucosa and skin microflora Immunological and cytological indicators Allergen test Adverse event UAS7 VAS itch score 5D itch scale DPS DLQI scale CU-Q2oL MOS-SS sleep scale Patient and observer satisfaction |
| Starting date | Not yet recruiting |
| Contact information | Guang Yin, Department of Dermatology, PLA General Hospital, 28 Fuxing Road, Haidian District, Beijing, China |
| Notes | Not yet recruiting |

**3.10.6 Moderate to severe chronic plaque psoriasis**

The registered RCT of moderate to severe chronic plaque psoriasis were shown in See Table S6.

Table S6 moderate to severe chronic plaque psoriasis (ChiCTR1900021307)

| Trial name or title | Clinical study for fecal microbiota transplantation in the treatment of moderate-to-severe chronic plaque psoriasis |
| --- | --- |
| Methods | Interventional (Clinical Trial), randomized |
| Participants | Inclusion criteria: (1) subjects will voluntarily participate in the study, sign a written informed consent, and comply with the study requirements; (2) male or female aged at least 18 years; (3) patients with moderate-to-severe chronic plaque psoriasis were defined as follows at baseline: BSA at least 10%, PASI at least 12; (4) poor control by local treatment and/or phototherapy and/or previous systemic treatment; (5) patients with chronic constipation for at least 6 months or chronic diarrhea for at least 2 months.  Exclusion criteria: (1) suffering from other skin diseases except psoriasis; (2) suffering from psoriasis other than plaque psoriasis; (3) suffering from drug psoriasis; (4) received nb-uvb treatment within two months or did not want to stop phototherapy during the study period; (5) used other external medicine treatment for psoriasis other than medical emollients within 1 month; (6) received systemic therapy for psoriasis (glucocorticoids, immunosuppressants, retinoic acid derivatives, etc.) within two months; (7) exposure to biological agents within 6 months; (8) is using other experimental drugs or is in 5 times half-life period or within 30 days (whichever is the older); (9) those who have received immunomodulator treatment within 1 year; (10) pregnant, pregnant and lactating women; (11) in addition to psoriasis, there is a history of other serious systemic progressive diseases (such as history of active inflammatory diseases, history of chronic or recurrent progressive infectious diseases, and history of malignant diseases, etc.); (12) concomitant:  (1) significant medical problems, including, but not limited to, uncontrolled hypertension (systolic blood pressure 160mmHg and/or diastolic blood pressure 95mmHg), congestive heart failure (New York heart association status class III or IV). The serum creatinine level exceeded 2.0mg/dl (176.8 mol/L).White blood cell count <2500/l or platelet <100000 / l or neutrophil <1500 /l or hemoglobin <8.5g/dl at the time of screening; (13) have used anti-microbial drugs, probiotics, intestinal microecological regulation therapy, high-dose vitamins and other pharmacological treatments that have a greater impact on intestinal flora within 1 month; (14) congenital immune deficiency or low immunity; (15) surgery for other diseases within 2 months or may be required; (16) for any other reason, the researcher considers it inappropriate to participate in this study. |
| Interventions | Intervention: FMT+external medical emollient Comparison: Gastrointestinal endoscopy and normal saline injection + external medical emollient |
| Outcomes | Primary outcomes: PASI and BSA SF-36 scale +DLQI scale adverse reaction Secondary outcomes: Structure and diversity of faeces, intestinal mucosa and skin microflora Immunological indicators Cytological index Skin histopathology and immunohistochemistry |
| Starting date | Not yet recruiting |
| Contact information | Guang Yin, Department of Dermatology, PLA General Hospital, 28 Fuxing Road, Haidian District, Beijing, China |
| Notes | Not yet recruiting |

**3.10.8 Multiple sclerosis**

The registered RCT of moderate to multiple sclerosis were shown in Table S7.

Table S7 multiple sclerosis

| NCT04300426 | Trial name or title | Safety and Efficacy of Anaerobic Cultivated Human Intestinal Microbiome Transplantation in Systemic Sclerosis (ReSScue) |
| --- | --- | --- |
|  | Methods | Interventional (Clinical Trial), randomized |
|  | Participants | Inclusion Criteria: 1. Participant must be 18 to 85 years of age inclusive, at the time of signing the informed consent. 2. Participants must have been clinically diagnosed with SSc by a rheumatologist having experience with the disease. 3. Participants must have disease characteristics that fulfill the 2013 ACR/EULAR classification criteria for SSc. 4. Participants must be able to understand and follow trial procedures including completion of questionnaires regarding Patient Reported Outcome measures, such as the Norwegian version of the UCLA GIT V2.0 score. 5. Participants must have moderate to severe SSc-related lower GI symptoms at time of inclusion, as defined by UCLA GIT score values of ≥1.01 for bloating and/or ≥0.50 for diarrhea at the screening visit. 6. Male and female 7. Capable of giving signed informed consent as described in Appendix 1 which includes compliance with the requirements and restrictions listed in the informed consent form (ICF) and in this protocol." Exclusion Criteria: Medical Conditions 1. Cardiovascular diseases, any of the following a. Severe hypertension, uncontrolled under treatment (≥160/100 mmHg), within 6 month of Visit 1 b. Myocardial infarction within 6 months of Visit 1 c. Unstable cardiac angina within 6 months of Visit 1 2. Lung disease with impaired respiratory function, any of the following a. Forced Vital Capacity (FVC) < 50% of expected reference value within 12 month of Visit 1 b. Diffusing lung capacity for carbon monoxide (DLCO) < 40% of expected reference value within 12 month of Visit 1 c. LTOT or lung-tx 3. Significant pulmonary hypertension, any of the following a. Previous clinical or echocardiographic evidence of significant right heart failure b. History of right heart catheterisation showing a cardiac index ≤ 2 l/min/m² 4. History of thrombotic event (including stroke and transient ischemic attack) within 12 months of Visit 1 5. Severe anemia with Hb < 8.0 g/l within 4 weeks prior to Visit 1. Repeat testing of Hb is allowed. 6. Bleeding risk, any of the following a. History of hemorrhagic central nervous system (CNS) event within 12 months of Visit 1. b. Known genetic predisposition to bleeding c. Platelet counts < 50 x 109/l 7. Chronic liver disease or gastro-intestinal condition, any of the following a. Primary biliary cholangitis b. Primary sclerosing cholangitis c. Decompensated chronic liver disease d. Inflammatory bowel disease e. Celiac disease treated for less than 12 months. 8. Gastro-intestinal surgery performed within the within 12 months of Visit 1 9. Hepatic dysfunction, as defined as AST, ALT or bilirubin levels >3 times the Upper limit of normal range (x ULN) within 4 weeks prior to Visit 1. Repeat testing of AST, ALT and bilirubin are allowed in participants with no prior history of hepatic dysfunction. 10. Chronic renal insufficiency, with estimated Glomerular Filtration Rate (eGFR) < 30. 11. Active digital ulcers within 4 weeks of Visit 1. 12. Anaphylactic food allergy. 13. Eating disorder diagnosed by a physician 14. Other diseases or conditions that may interfere with testing procedures (for example inability to conduct gastroduodenoscopy) or in the judgment of the Investigator may interfere with trial participation or may put the patient at risk when participating in this trial (for example severe GI symptoms due to other diseases than SSc). Prior/Concomitant Therapy 15. Any antibiotic therapy within 3 months of Visit 1 16. Prednisone >10 mg/day or equivalent within 4 weeks prior to Visit 1 17. Cyclophosphamide or rituximab treatment within 6 months prior to Visit 1 18. Unstable background monotherapy with any of the following therapeutics; mycophenolate mofetil/sodium, methotrexate, azathioprine, tocilizumab, abatacept, leflunomide, tacrolimus, tofacitinib and cyclosporine A. Participants have to be on stable monotherapy with any of these medications for at least 6 months prior to visit 1 19. Combined therapy of two or more of the following therapeutics: mycophenolate mofetil/sodium, methotrexate, azathioprine, tocilizumab, abatacept, leflunomide, tacrolimus, tofacitinib and ciclosporine A within at least 8 weeks prior to visit 1. 20. Need for full-dose therapeutic anticoagulation (e.g. vitamin K antagonists, direct thrombin inhibitors or heparin) 21. Previous hematopoietic stem cell transplantation (HSCT) within 12 months of Visit 1, or HSCT planned within 12 months after Visit 1. 22. Other investigational therapy received within 1 month or 6 half-lives (whichever was greater) prior to screening visit. Prior/Concurrent Clinical Study Experience 23. Prior participation in FMT study in the last 12 months. Diagnostic assessments 24. Abnormal coagulation parameters as defined as International normalised ratio (INR) >2, prolongation of prothrombin time (PT) and partial thromboplastin time (PTT) by >1.5 x ULN at Visit 1 Other Exclusions 25. Women who are pregnant, nursing, or who plan to become pregnant while in the trial. (Women of child bearing potential should be tested with Hcg (urine or serum). Woman of child bearing potential if not using highly efficient contraception. |
|  | Interventions | Intervention: "ACHIM" as solute (10^9 intestinal microbes/ml) Comparison: ACHIM culture media (no bacteria) |
|  | Outcomes | Primary Outcome Measures:  1. Change from baseline to week 12 in UCLA GIT score item diarrhea or bloating, depending Secondary Outcome Measures : 1. Adverse event 2. Change from baseline to week 12 in total UCLA GIT score 3. Change from baseline to week 12 in UCLA GIT score item diarrhea 4. Change from baseline to week 12 in UCLA GIT score item bloating Other Outcome Measures: 1. Change from baseline to week 12 in Faecal incontinence quality of life scale 2. Change from baseline to week 12 in UCLA GIT score item reflux 3. Change from baseline to week 12 in UCLA GIT score item fecal soilage 4. Change from baseline to week 12 in UCLA GIT score item constipation 5. Change from baseline to week 12 in UCLA GIT score item emotional wellbeing  6. Change from baseline to week 12 in UCLA GIT score item social participation 7. Change from baseline to week 12 in Change from baseline to week 12 in HAQ-DI scores 8. Change from baseline to week 12 in VAS Fatigue scale 9. Change from baseline to week 12 in ScleroId score 10. Change from baseline to weeks 2, 6 and 12 in overall faecal microbiome composition measured by 16sRNA based methods 11. Change from baseline to week 12 in saliva, skin and urine microbiome measured by 16sRNA based methods 12. Change from baseline to weeks 2, 6 and 12 in immunoglobulin bound fraction of the overall faecal microbiome 13. Change from baseline to week 12 in gastrointestinal transit time and contractions evaluated by SmartPill technology along with registration of stool frequency and consistency by Bristol Stool Scale 14. Change from baseline to weeks 6 and 12 in peripheral blood B cell and T cells (as evaluated by receptor sequencing, proteomics and cellular phenotyping) and content of soluble molecules 15. Change from baseline to weeks 2 and 12 in the architecture and cellular composition of duodenal biopsy specimens (including characterization of cellular surface markers, proteomics, metabolomics and immune cell receptor sequencing 16. Change from baseline to week 12 in skin properties evaluated by elastography and ultrasonographic skin thickness 17. Change from baseline to week 12 in Health-related Quality of Life assessed by EQ-5D  18. Change from week 12 through week 20 in all participants, and up to week 36 in a subset of participants in UCLA GIT score item diarrhea or bloating, depending which was the worst symptom at the baseline evaluated separately for each patient 19. Follow changes of UCLA total GIT score from week 12 through week 20 in all participants, and up to week 36 in a subset of participants 20. Follow changes in mean of HAQ-DI; VAS Fatigue; ScleroId score; and patient reported global assessment from week 12 through week 20 in all participants, and up to week 36 in a subset of participants 21. Assess changes of overall faecal microbiome composition measured by 16sRNA based methods from week 12 through week 20 in all participants, and up to week 36 in a subset of participants  22. Assess change of saliva, skin and urine microbiome measured by 16sRNA based methods from week 12 through week 20 in all participants 23. Assess change in peripheral blood B cell and T cells and content of soluble molecules from week 12 through week 20 in all participants, and up to week 36 in a subset of participants 24. Assess changes in upper GIT scores from week 12 to 20 and determine potential associations to the architecture and cellular composition of oesophagus biopsy specimens 25. Assess change from week 12 to 20 in Health-related Quality of Life assessed by EQ-5D from week 12 through week 20 in all participants, and up to week 36 in a subset of participants |
|  | Starting date | September 24, 2020 |
|  | Contact information | Anna-Maria Hoffmann-Vold, MD, PhD Oslo University Hospital, Oslo, Norway |
|  | Notes | Recruiting; Estimated Enrollment: 70 |
| ChiCTR1800018057 | Trial name or title | A randomized controlled clinical trial of fecal bacteria transplantation for multiple sclerosis |
|  | Methods | Interventional (Clinical Trial), randomized |
|  | Participants | Inclusion criteria: (1) Aged 18-55 years; (2) MS is diagnosed according to the diagnostic criteria of McDonald (Revised Edition 2010); (3) the course of the disease was 2-10 years; (4) the baseline of the EDSS score was 2-5 points before the treatment; (5) relapse remission has recurred in the past 12 months. (6) secondary progressive type had recurrence within the past 12 months, or the EDSS score increased by 1 points. (7) there were recurrences in primary progression or GAD enhancement in head MRI.  Exclusion criteria: (1) diseases that interfere with clinical assessment, such as disability (not caused by MS); (2) severe depression; (3) pregnant, lactating women or those who are planning to conceive; (4) do not agree to sign the patient's informed consent; (5) relapse within 30 days. (6) antibiotic treatment in the last 30 days |
|  | Interventions | Intervention: fecal microbiota transplantation Comparison: conventional therapy |
|  | Outcomes | Primary Outcome Measures:  The EDSS increased by 1 points or more in 60 weeks and continued for 12 weeks. Secondary Outcome Measures: The progression of upper limb functional disability increased by 20% at 60 weeks and continued for 12 weeks. the ratio of 25 step walking speed of the subjects decreased by 20% and lasted for 12 weeks in 60 week. Recurrence times Annual recurrence rate new lesion in MRI Adverse events |
|  | Starting date | Not yet recruiting |
|  | Contact information | Zhou Liu, Affiliated Hospital of Guangdong Medical University, 57 People's Avenue, Xiashan District, Zhanjiang, Guangdong, China |
|  | Notes | Not yet recruiting |
