# Supplementary material for: Safety and efficacy of fecal microbiota transplantation for autoimmune diseases and autoinflammatory diseases: A systematic review and meta-analysis
Source: Front Immunol. 2022 Sep 30;13:944387. doi: 10.3389/fimmu.2022.944387 (PMC9562921; doi:10.3389/fimmu.2022.944387)
Supplement: Supplementary file 4 [file Table_4.docx]

**3.11 Other registered non-RCTs related to autoimmune diseases**

In addition, after searching in ClinicalTrials.gov and the Chinese Clinical Trials Registry, a total of registered non-RCTs for FMT treatment of 5 types of autoimmune diseases were found: Refractory IgA nephropathy, primary sclerosing cholangitis, multiple sclerosis, T1DM, gout. The characteristics of ongoing studies were shown in Table S8-S12.

**3.11.1 Refractory IgA nephropathy**

The registered clinical trials of Refractory IgA nephropathy were shown in Table S8.

Table S8 Refractory IgA nephropathy

| NCT03633864 | Trial name or title | Fecal Microbiota Transplantation for Refractory IgA Nephropathy |
| --- | --- | --- |
|  | Methods | Interventional (Clinical Trial, Cohort) |
|  | Participants | Inclusion Criteria: Adult, age: 18-65 years old. Pathological diagnosis with IgAN, eGFR：20-120 mL/min/1.73 m2. The 24-hour urinary protein was still greater than 1g after 3-6 months of treatment of ACEI/ARB Recurrence of IgAN after glucocorticoid and (or)immunosuppressant decrement and the 24-hour urinary protein was greater than 1g. Not suitable for the administration of glucocorticoid and (or) immunosuppressive agents because of side effects. Women of child-bearing age with negative urine pregnancy test have no pregnancy plan for the next 18 months and take effective contraceptive measures. Agree to participate in this clinical trial Exclusion Criteria: Malignant tumors and other diseases with expected survival time <3 months. Severe cardiovascular and cerebrovascular diseases and pulmonary dysfunction. Other immune system diseases. Diabetes Inflammatory bowel disease（IBD） Clostridium difficile infection Gastrointestinal tumor Active gastrointestinal bleeding Acute and chronic gastroenteritis Have received or are receiving FMT treatment. HIV Psychosis AND dysgnosia Contraindication of colonoscopy and enema Alcohol/drug abuse Other conditions that the researchers thought were not appropriate for the group. |
|  | Interventions | FMT |
|  | Outcomes | Primary Outcome Measures  : 1. Change of Urinary protein Secondary Outcome Measures  : 1. Change of Serum creatinine 2. Change of eGFR 3. Change of Hematuria 4. Change of Blood pressure 5. Change of Serum IgA1 6. Change of Fecal microbiota 7. Adverse events associated with FMT |
|  | Starting date | November 22, 2017 |
|  | Contact information | Shiren Sun, M.D., Xijing Hospital of Nephrology, Xi'an, Shaanxi, China, 710032 |
|  | Notes | Unknown; Estimated Enrollment: 30 |

**3.11.2** **Primary sclerosing cholangitis**

The registered clinical trials of primary sclerosing cholangitis were shown in Table S9.

Table S9 primary sclerosing cholangitis

| NCT02424175 | Trial name or title | Fecal Microbiota Transplantation for the Treatment of Primary Sclerosing Cholangitis |
| --- | --- | --- |
|  | Methods | Interventional (Clinical Trial) |
|  | Participants | Inclusion Criteria: Age 18 or older Confirmed diagnosis of PSC (with a concurrent diagnosis of inflammatory bowel disease) characterized by a cholestatic liver condition of greater than 6 months duration with confirmatory cholangiographic findings, as well as an elevation of the serum alkaline phosphatase of greater than 1.5 times the upper limit of normal. Exclusion Criteria: Decompensated liver disease Patients who were pregnant or breastfeeding Use of concomitant immune modulators including methotrexate, mycophenolate mofetil, tacrolimus, cyclosporine, thalidomide, Interleukin-10, or Interleukin-11 within 4 weeks prior to receiving the FMT Patients who are unable to give informed consent Patients who are unable or unwilling to undergo colonoscopy with moderate sedation (>ASA class II) Patients who have previously undergone FMT Patients who have a confirmed malignancy or cancer Patients who are immunocompromised Treatment within last 8 weeks with infliximab, adalimumab, certolizumab, natalizumab, vedolizumab or thalidomide Antibiotic use within 2-months of start date Participation in a clinical trial in the preceding 30 days or simultaneously during this trial Probiotic use within 30 days of start date Congenital or acquired immunodeficiencies Other comorbidities including:Diabetes mellitus, cancer, systemic lupus, must be able to tolerate conscious sedation with colonoscopy Chronic kidney disease as defined by a GFR <60mL/min/1.73m2 44 History of rheumatic heart disease, endocarditis, or valvular disease due to risk of bacteremia from colonoscopy Steroid dose >20mg/day |
|  | Interventions | FMT |
|  | Outcomes | Primary Outcome Measures: Adverse Event Frequency Number of patients with reporting adverse events irregardless of severity Comparison of Alkaline Phosphatase Pre and Post Transplant Secondary Outcome Measures : Microbiome |
|  | Starting date | February 1, 2016 |
|  | Contact information | Joshua Korzenik, Director, Crohn's and Colitis Center, Brigham and Women's Hospital, Boston, Massachusetts, United States, 02115 |
|  | Notes | Completed in May 8, 2018; Actual Enrollment: 10 |

**3.11.3** **Multiple sclerosis**

The registered clinical trials of multiple sclerosis were shown in Table S10.

Table S10 multiple sclerosis

| NCT03975413 | Trial name or title | Fecal Microbiota Transplantation (FMT) in Multiple Sclerosis |
| --- | --- | --- |
|  | Methods | Interventional (Clinical Trial, case report) |
|  | Participants | Inclusion Criteria: Older than 18 years of age. Diagnosis of relapsing-remitting multiple sclerosis (RRMS) by neurology(primary specialist). Presence of active lesions on brain or spinal cord MRI, in the past 1 year prior to baseline. MS disease duration greater than 1 year. Symptomatic (Active RRMS). On MS therapy/medication greater than 4 weeks. Exclusion Criteria: Newly diagnosed multiple sclerosis. Inactive relapsing-remitting multiple sclerosis (RRMS). Unstable or no MS therapy/medication use. Presence of symptomatically active gastrointestinal diseases such as inflammatory bowel disease or celiac disease (except for hemorrhoids, hiatal hernia, or occasional (˂3 times a week) heartburn)). Pre-existent organ failure or co-morbidities as these may change GI flora: a) liver disease (cirrhosis or persistently abnormal AST or ALT that are 2X˃ normal); b) kidney disease (creatinine ˃ 2.0mg/dL); c) uncontrolled psychiatric illness; d) clinically active lung disease or decompensated heart failure; e) known HIV infection; f) alcoholism; g) transplant recipients (other than FMT); h) diabetes Severe malnutrition or obesity with BMI ˃ 40. Antibiotic and probiotic use (except yogurt) within 4 weeks of enrollment. Chronic use of NSAIDS. A washout period of 3 weeks is needed before the subject could be enrolled into the study. Low dose aspirin is allowed. Pregnant or lactating women or intention of getting pregnant during the trial period. Active infection including untreated latent or active tuberculosis, HIV, hepatitis, syphilis or other major active infection. Active symptomatic C. Difficile infection (colonization is not an exclusion). Active gastrointestinal condition being investigated (i.e. GI bleeding, colon cancer, active GI workup); history of known or suspected toxic megacolon and/or known small bowel ileus, major gastrointestinal surgery (e.g. significant bowel resection) within 3 months before enrollment (note that this does not include appendectomy or cholecystectomy); or history of total colectomy or bariatric surgery. |
|  | Interventions | FMT |
|  | Outcomes | Primary Outcome Measures: Fecal microbial community structure and functional changes over six time frames for phylum, genus and species taxonomic level bacteria, virus, fungi, and archaea. Walking and balance changes over four time frames for stride time (seconds). Walking and balance changes over four time frames for stride distance (meters).  Walking and balance changes over four time frames for cadence (total number of steps per minute). Walking and balance changes over four time frames for step width (meters). Walking and balance changes over four time frames for average pelvis forward velocity (meters per second). Walking and balance changes over four time frames for pelvis smoothness (pelvis horizontal speed). Secondary Outcome Measures: Fecal targeted short-chain-fatty-acid metabolomics concentration changes over six time frames for acetate (mM/kg), propionate (mM/kg), butyrate (mM/kg), and total SCFA (mM/kg). Measurement of blood serum biomarker brain-derived neurotrophic factor (BDNF) (ng/ml) changes over six time frames. Sleep changes over six time frames. Food timing changes over six time frames. Gastrointestinal symptoms changes over six time frames (t-scores, mean, standard deviations). Walking changes over six time frames. Lesions changes over three time frames.  Food and frequency of consumption changes over six time frames. Single day food recall changes over six time frames. Diet changes over six time frames. Measurement of blood serum biomarker Interleukin-6 (IL-6) (pg/ml) changes over six time frames. Measurement of blood serum biomarker Interleukin-* (IL-8) (pg/ml) changes over six time frames. Measurement of blood serum biomarker Tumor necrosis factor alpha (TNFα) (pg/ml) changes over six time frames. |
|  | Starting date | September 25, 2018 |
|  | Contact information | Ali Keshavarzian, MD Rush University Medical Center, Chicago, Illinois, United States, 60612 |
|  | Notes | Completed in May 1, 2020; Actual Enrollment: 1 |
| NCT03594487 | Trial name or title | Fecal Microbiota Transplantation (FMT) of FMP30 in Relapsing-Remitting Multiple Sclerosis (MS-BIOME) |
|  | Methods | Interventional (Clinical Trial) |
|  | Participants | Inclusion Criteria: Age 18-60 inclusive (at time of screening). Diagnosis of relapsing-remitting multiple sclerosis (MS) by International Panel McDonald Criteria (2010)(1) incorporating 2017 revisions which reclassify select high-risk Clinically Isolated Syndromes under 2010 criteria as RRMS under 2017 criteria, and Lublin criteria (2014)(2). Recent documented MS disease activity, defined as at least 1 clinical relapse within the past 1 year prior to baseline OR 2 clinical relapses in the past 2 years prior to baseline OR at least 1 new T2/FLAIR lesion on brain or spine MRI OR at least 1 gadolinium enhancing lesion on brain or spine MRI in the past 1 year prior to baseline. Expanded Disability Status Scale (EDSS) less than or equal to 6.0; EDSS 5.5 or less if MS disease duration is greater than 15 years (no other disease duration restriction). Must have positive serology for Epstein-Barr Virus (EBV) (IgG anti-EBNA positive) at screening, indicating prior exposure. No prior MS disease modifying therapy or a 12 week washout period for subjects on glatiramer acetate or interferon-beta therapy. At least 4 weeks from baseline since last use of IV or oral glucocorticoids Protocol: MS-BIOME Study. Agree to maintain a stable diet during the course of the study (over the counter probiotics are allowable). Premenopausal women and women <12 months after the onset of menopause must have a negative serum pregnancy test unless they have undergone surgical sterilization. Female subjects of childbearing potential who are sexually active with a non-sterilized male partner must agree to use a highly effective method of contraception; non-sterilized male subjects who are sexually active with a female partner of childbearing potential must agree to use a highly effective method of contraception. Not actively participating in another interventional MS clinical trial (participation in other observational research studies is allowable). Exclusion Criteria: Prior use of fingolimod, dimethyl fumarate, teriflunomide, natalizumab, alemtuzumab, mitoxantrone, cyclophosphamide, rituximab, ocrelizumab, daclizumab, methotrexate, azathioprine, mycophenolate mofetil, cyclosporine, leflunomide or induction chemotherapy. No use of diuretics like furosemide (Lasix) 1 week before the first dose oral antibiotics. The use of hydrochlorothiazide (HCTZ) for hypertension at a dose < 50 mg/day is allowable. Progressive MS by Lublin criteria (2014). No oral or IV antibiotics within 8 weeks of screening and 12 weeks of scheduled of the planned FMT procedure if in the FMT arm or first stool collection if in control arm (note that topical, otic, ocular antibiotics are specifically allowable which is consistent with the IMSMS.org protocol for collaborative gut microbiome research in MS). Hypersensitivity or allergy to study antibiotics, conscious sedation medications or bowel preparation. Contraindication to study procedures including MRI, anesthesia (ASA criteria IV and V), colonoscopy, phlebotomy. History of inflammatory bowel disease (Crohn's Disease, Ulcerative Colitis) Protocol: MS-BIOME Study. Active symptomatic C. Difficile infection (colonization is not an exclusion). Active gastrointestinal condition being investigated (i.e. GI bleeding, colon cancer, active GI workup); history of known or suspected toxic megacolon and/or known small bowel ileus, major gastrointestinal surgery (e.g. significant bowel resection) within 3 months before enrollment (note that this does not include appendectomy or cholecystectomy); or history of total colectomy or bariatric surgery. History of malignancy (except excised cutaneous basal cell carcinoma or squamous cell carcinoma which are allowable) including no concurrent induction chemotherapy, radiation therapy or biological treatment for active malignancy. Pregnant or lactating women or intention of getting pregnant during the trial period. Active infection including untreated latent or active tuberculosis, HIV, hepatitis, syphilis or other major active infection. Known immunodeficiency including CVID. INR>1.5, Platelets<100, Hemoglobin <8.5, WBC<2.0, Absolute lymphocyte count <0.8, Absolute Neutrophil Count <0.5, CD4<200, eGFR<45. Any condition that in the opinion of the study PI could jeopardize the safety of the subject, would make it unlikely for the subject to complete the study or could confound the results of the study. Unable or unwilling to comply with study protocol requirements. |
|  | Interventions | Intervention: FMP30 Donor Stool Comparison: Observational Control |
|  | Outcomes | Primary Outcome Measures: Subjects who complete the study protocol Change in fecal microbiota Incidence of Treatment-Emergent Adverse Events Secondary Outcome Measures: Induction of T regulatory or Th2 cells and/or reduction of Th1 or Th17 cells Plasma CD19+ B cell count percentages Plasma CD20+ B cell count percentages Plasma CD19+ B cell absolute count Plasma CD20+ B cell absolute count Measurement of Serum Immunoglobulin Levels Incidence of new T2/FLAIR lesions Measurement of T2/FLAIR lesion volume Number of T2/FLAIR lesions Total Gadolinium Enhancing Lesions |
|  | Starting date | November 16, 2018 |
|  | Contact information | Jeffrey Gelfand UCSF Multiple Sclerosis Center, San Francisco, California, United States, 94158 |
|  | Notes | Active, not recruiting; Estimated Enrollment: 30 |
| NCT04203017 | Trial name or title | Fecal Microbiota Transplantation After Autologous HSCT in Patients With Multiple Sclerosis |
|  | Methods | Interventional (Clinical Trial) |
|  | Participants | Inclusion Criteria: Diagnosis: Multiple sclerosis (Relapsing-Remitting, Secondary-Progressive, Primary-Progressive) AutoHSCT Signed informed consent No second tumors No severe concurrent illness 1.0-6.5 points by EDSS Disease duration less than 20 years Disease progression on 1 and/or 2 line therapy (1 point EDSS 1.0-6.0 and 0,5 point EDSS 6.0-6.5) Exclusion Criteria: Moderate or severe cardiac dysfunction, left ventricular ejection fraction <50% Moderate or severe decrease in pulmonary function, FEV1 <70% or DLCO<70% of predicted Respiratory distress >grade I Severe organ dysfunction: AST or ALT >5 upper normal limits, bilirubin >1.5 upper normal limits, creatinine >2 upper normal limits Creatinine clearance < 60 mL/min Uncontrolled bacterial or fungal infection at the time of enrollment Requirement for vasopressor support at the time of enrollment Karnofsky index <30% Pregnancy Somatic or psychiatric disorder making the patient unable to sign informed consent |
|  | Interventions | Allogeneic FMT |
|  | Outcomes | Primary Outcome Measures: To evaluate effectiveness of autoHSCT in combination with FMT in patients with refractory multiple sclerosis Secondary Outcome Measures: To evaluate overall survival after autoHSCT in combination with FMT in patients with refractory multiple sclerosis To evaluate adverse effects after FMT in immunocompromised patients Quality of life status 1 Quality of life status 2 Evaluation of Immune system reconstitution after autoHSCT 1 Evaluation of Immune system reconstitution after autoHSCT 2 Impact of autoHSCT on brain structure anatomy |
|  | Starting date | June 1, 2019 |
|  | Contact information | Boris Afanasyev, Professor Pavlov First Saint-Petersburg State Medical University, Saint Petersburg, Russian Federation, 197022 |
|  | Notes | Recruiting; Estimated Enrollment: 20 |

**3.11.4** **T1DM**

The registered clinical trials of T1DM were shown in Table S11.

Table S11 T1DM

| NCT04124211 | Trial name or title | Fecal Microbiome Transplantation (FMT) for Type 1 Diabetes |
| --- | --- | --- |
|  | Methods | Interventional (Clinical Trial) |
|  | Participants | Inclusion Criteria: (1) Type 1 diabetes patients. (2) Age between 18 and 65 years old, regardless of gender. (3) No serious comorbidities. (4) Accept and suitable for endoscopic catheterization (TET) and fecal transplantation (FMT). (5) Can receive follow-up and follow-up examinations on time. (6) Subjects need to sign an informed consent form. Exclusion Criteria: (1) Systematic application of glucocorticoids, other immunosuppressive drugs or biological immune modulators, antibiotics, probiotics, and other microecological agents to alter intestinal motility within 6 months prior to enrollment. (2) An infection that is active. (3) Combined with irritable bowel syndrome, inflammatory bowel disease, celiac disease, and other chronic gastrointestinal diseases, the condition has not been controlled. (4) Chronic diseases such as cerebrovascular disease, cardiovascular disease, and diabetic autonomic neuropathy. (5) Pregnancy or with a pregnancy plan (6) severe organ dysfunction (including decompensated cirrhosis, malignant tumors, etc.) |
|  | Interventions | FMT |
|  | Outcomes | Primary Outcome Measures: Changes in mean amplitude of glycemic excursion (MAGE) Changes in standard deviation of blood glucose (SDBG) Changes in hemoglobin A1c (HbA1c) Safety of FMT Secondary Outcome Measures: Changes in 24h mean blood glucose(MBG) Changes in percentage of time of blood glucose(PT) Changes in mean absolute glucose(MAG) Changes in standard deviation of blood glucose(SDBG) Changes in coefficient of variation(CV) Changes in high blood glucose index(HBGI) Changes in low blood glucose index(LBGI) Changes in effective blood glucose fluctuations in frequency(NGE) Changes in glycated albumin (GA) Changes of serum C-peptide (fasting, 30min after meal, 120min after meal) Assessment of diabetes antibodies Changes in intestinal microbiome profile Changes in Peripheral Blood Stem Cell (PBMC) Changes in body weight to calculate body mass index (BMI) Pathological changes of intestinal mucosa Changes in blood pressure Changes in oral mucosal bacteria colonization Changes in urine microalbumin Blood chemistry panel |
|  | Starting date | August 25, 2019 |
|  | Contact information | Jie Shen, MD The Third Affiliated Hospital of Southern Medical University, Guangzhou, Guangdong, China, 510630 |
|  | Notes | Recruiting; Estimated Enrollment: 10 |

**3.11.5** **Gout**

The registered clinical trials of gout were shown in Table S12.

Table S12 Gout

| ChiCTR2000034584 | Trial name or title | Clinical study on the effects of fecal bacteria transplantation on uric acid and intestinal barrier function in gout patients |
| --- | --- | --- |
|  | Methods | Interventional (Clinical Trial) |
|  | Participants | Inclusion criteria: 1. aged >=18 years; 2. diagnosed with gout according to the Clinical Gout Diagnosis Criteria[12]; 3. treated with uric-acid-lowering drugs for more than one year, but still with hyperuricemia and suffering from acute gout flares twice or more a year.  Exclusion criteria: 1. pregnancy; 2. gastrointestinal infection, cardiopulmonary failure or serious liver diseases; 3. rejection to conduct transendoscopic enteral tubing. |
|  | Interventions | Intervention: FMT to Hyperuricemia patients with gout Comparison: Hyperuricemia patients without gout |
|  | Outcomes | Outcomes: Uric acid Intestinal barrier Symptoms |
|  | Starting date | Not known |
|  | Contact information | Xie Wenrui, The First Affiliated Hospital of Guangdong Pharmaceutical University, 19 Nonglin Road Down, Guangzhou, Guangdong, China |
|  | Notes | None |
